# A Longitudinal Analysis of the Association Between Long-Term Exposure to Air Pollution and Cognitive Function Among Adults Aged 45 and Older in China

**DOI:** 10.1093/geronb/gbac162

**Published:** 2022-10-10

**Authors:** Kai Hu, Jo Mhairi Hale, Hill Kulu, Yang Liu, Katherine Keenan

**Affiliations:** Department of Sociology, East China University of Science and Technology, Shanghai, 200237, China; Population and Health Research Group, School of Geography and Sustainable Development, University of St Andrews, Fife, UK; Population and Health Research Group, School of Geography and Sustainable Development, University of St Andrews, Fife, UK; Population and Health Research Group, School of Geography and Sustainable Development, University of St Andrews, Fife, UK; Gangarosa Department of Environmental Health, Rollins School of Public Health, Emory University, Atlanta, Georgia, USA; Population and Health Research Group, School of Geography and Sustainable Development, University of St Andrews, Fife, UK

**Keywords:** Air pollution, Cognitive function, Cumulative exposure, Health disparities, PM_2.5_

## Abstract

**Objectives:**

Evidence suggests long-term exposure to fine particulate matter air pollution (PM_2.5_) is associated with a higher risk of cognitive impairment, especially among older adults. This study examines the relationship between PM_2.5_ exposure and cognitive function in China’s aging population.

**Methods:**

We used longitudinal data from the China Health and Retirement Longitudinal Study (2011–2015) linked with historical PM_2.5_ concentrations (2000–2015) from remotely sensed satellite data. Growth curve models were applied to estimate associations between PM_2.5_ exposure (measured in intensity, duration, and a joint variable of intensity with duration for cumulative exposure) and cognitive function.

**Results:**

Relative to the lowest exposure group, exposure in the second group of PM_2.5_ intensity (35–50 μg/m^3^) is associated with poorer cognitive function, but higher levels of PM_2.5_ appear to be associated with better cognitive function, indicating a U-shaped association. Similar patterns are seen for fully adjusted models of PM_2.5_ duration: the second group (13–60 months) is associated with worse cognitive function than the first group (0–12 months), but coefficients are nonsignificant in longer duration groups. Joint analysis of PM_2.5_ intensity with duration suggests that duration may play a more detrimental role in cognitive function than intensity. However, we do not find a statistically significant association between PM_2.5_ exposure and the rate of cognitive decline.

**Discussion:**

Our findings are mixed and suggest that some categories of higher and longer exposure to PM_2.5_ are associated with poorer cognitive function, while that exposures do not hasten cognitive decline. However, more work is necessary to disentangle PM_2.5_ exposure from individuals’ background characteristics, particularly those jointly associated with cognitive function and urban living.

Cognitive impairment reduces healthy life expectancy, affects the quality of later life, and contributes substantially to health care burdens for individuals and societies (Clifford et al., 2016; Yao et al., 2022). Globally, 40% of dementia cases are related to 12 main modifiable risk factors, which include ambient air pollution (Calderón-Garcidueas et al., 2012; Fu & Yung, 2020; Livingston et al., 2020; Peters et al., 2019; Wang et al., 2020). There are emerging population-based studies of the association between fine particulate matter smaller than 2.5 micrometers (μm) aerodynamic diameter (PM_2.5_) in outdoor air pollution and cognitive impairment and other neurological diseases (Ailshire & Clarke, 2015; Russ et al., 2019; Wang et al., 2020). PM_2.5_ consists of complex and varying mixtures of particles suspended in the air. Once inhaled, these particles can lead to systemic inflammation and oxidative stress across the blood–brain barrier (Block & Calderón-Garcidueñas, 2009). As a result, PM_2.5_ is a risk factor for declining brain function (Guxens & Sunyer, 2012).

China—one of the fastest-aging countries—experiences severe air pollution, where more than 50% of cities recorded PM_2.5_ levels far exceeding the World Health Organization safe levels in 2013 (Lancet, 2014). Air pollution in China is a result of the rapid industrial expansion occurring since 1979, which caused increases in coal consumption, motor, and industrial dust (Xu et al., 2013). The lives of those born before or during the 1970s have coincided with a period of rapid economic development, so they have spent large parts of their adult life course exposed to hazardous air pollution. For urban dwellers aged 45 and older, the long-term damage of air pollution is likely to be substantial (Lu et al., 2020). The effects of air pollution on health outcomes may vary by the age of individuals at exposure, because both children and older individuals are especially susceptible to deleterious air pollution (Peled, 2011; Shier et al., 2019). Research shows that exposure to PM_2.5_ is significantly associated with cognitive function in older adults (Ailshire & Clarke, 2015; Ailshire et al., 2017; Wang et al., 2020). Therefore, the aging and air pollution context in China is conducive to understanding the association between air pollution and cognitive function in more detail.

Although we have some knowledge about the association between exposure to severe air pollution and cognitive function, there remain some uncertainties. First, some studies, which have found that cognitive function is associated with air pollution exposure (Chen et al., 2017; Wang et al., 2020), have used average concentrations of air pollutants (mainly measured by the annual average) that do not completely capture effects of intensity and duration. Second, other studies use data that do not cover a long-term exposure window (Power et al., 2011; Sun & Gu, 2008). Thus, using multiple measures for exposure to air pollution over a long-term period (e.g., more than 10 years) is necessary to explore the association between time-integrated exposures and health risks.

In this study, we analyzed survey data from a large, prospective, nationally-representative cohort of Chinese adults ranging from 45 to 105 years of age, linked with 15 years of historical satellite data on PM_2.5_ exposure. We used growth curve models (GCM) to study the associations between PM_2.5_ exposure and cognitive function, comparing different ways of measuring PM_2.5_ exposure: intensity, duration, and a measure that takes into consideration both exposure intensity and its duration, which we called “cumulative exposure.”

## Method

### Study Population

Data were from three waves of the China Health and Retirement Longitudinal Study (CHARLS 2011–2015), which is a nationally representative longitudinal survey of the middle-aged and older population of China, consisting of persons 45 years of age or older, as well as their spouses when possible. The CHARLS used computer-assisted in-person interviews to obtain samples through four-stage stratified sampling, with an overall response rate of 80.5% at the baseline. From June 2011 to March 2012, the CHARLS conducted a baseline survey that included assessments of the social, economic, and health circumstances of 17,705 respondents from 28 provinces, 150 cities/counties/districts, and 10,257 households (Zhao et al., 2014). Following this baseline survey, two follow-up surveys were conducted in 2013 and 2015.

In 2011, the baseline CHARLS sample size was 17,705. Between 2011 and 2013, 3,130 respondents were lost due to death (*n* = 441) or nonspecified reasons (*n* = 2,509). In 2013, the CHARLS, to maintain age representation, added a refreshment sample of individuals who entered age eligibility of being 45 years and older between waves 1 and 2. If the baseline respondent shared a household with someone aged 40 and 44, he or she was reserved for a refreshment sample for future survey rounds. In wave 2, respondents who were aged 43–44 in wave 1 (plus their spouses) were added from the refreshment sample, the same for wave 3 in 2015, out of those aged 41–42 in wave 1. In wave 2 of the CHARLS 2013 (*n* = 18,064), 4,029 refreshment respondents were selected from household members of the original wave 1 respondent. Using the same strategy, the third wave of CHARLS in 2015 included 3,275 new individuals. Between 2013 and 2015, 689 respondents died, and by 2015, 809 respondents, interviewed in 2011 but missing in 2013, returned (*n* = 21,100).

**Figure 1.**
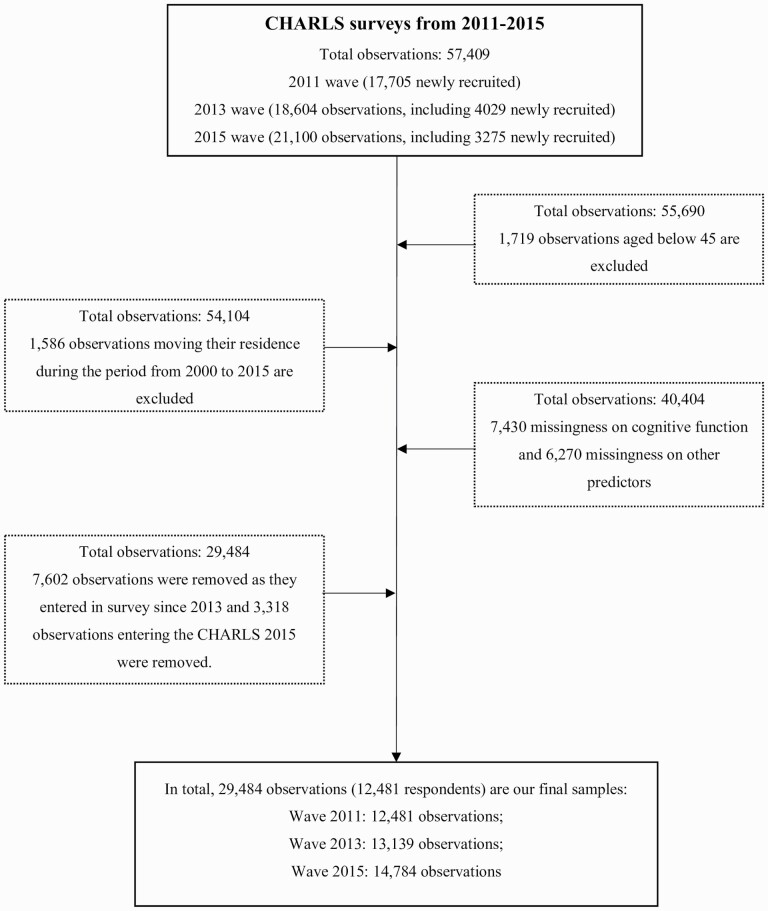
Flowchart of study inclusion criteria.

*Notes*: CHARLS = China Health and Retirement Longitudinal Study.

Figure 1 describes how we selected the analytical samples. First, we excluded 1,719 observations that were under the age of 45. The CHARLS did not collect detailed residential histories, so if respondents moved, it was difficult to reconstruct their air pollution exposure history. However, respondents self-reported the date they moved to their current residence, allowing us to exclude those who reported moving residence from 2000 to baseline or during the panel. Only 246 respondents (0.45% of the total samples) changed their residence in 2011, and 427 (0.79% of the total) and 913 (1.7% of the total) respondents moved in 2013 and 2015. We used listwise deletion for missingness on cognitive function (7,430 deleted) and other predictors (6,270 deleted). In order to make the exposure window consistent (see Supplementary Table S12), we have restricted the analytical sample to those individuals who entered the survey in 2011 (2013 and 2015 entrants were removed). Finally, our statistical analysis contains 29,484 observations (12,481 respondents) from 125 cities.

As a robustness check, we employed multiple imputation (MI) using chained equations, including all predictors that were in the analytical model (Carpenter & Kenward, 2012; van Ginkel et al., 2020; White et al., 2011; full details in Supplementary Material and Supplementary Tables S1 and S2). After creating multiple imputed data sets, the analysis was rerun, and the resulting models were combined using Rubin’s rules (Rubin, 1976), which take into account variation both within and between data sets.

### Outcome: Cognitive Function

Cognitive function was measured in the CHARLS by the modified Telephone Interview for Cognitive Status Survey, which includes orientation (recalling the date [year, month day], day of the week, and season of the year, 0–5 points), numeric ability (the serial subtraction of seven from 100, up to five times, 0–5 points), word recall (immediately repeating in any order 10 Chinese nouns, 0–10 points, and recalling the same list of words 4 min later, 0–10 points), and visuospatial ability (drawing a pentagon, 0–1 points). Following previous studies (Crimmins et al., 2011; Huang & Maurer, 2019; Kesavayuth et al., 2018), we used only variables reflective of fluid cognitive function (immediate and delayed word recall and serial 7s), which are considered more indicative of neurophysiological health rather than highly related to education and other sociocultural factors (Ghisletta et al., 2012). The range of fluid cognitive function in this study is from 0 to 25, with higher scores indicating better cognitive function.

### Air Pollution: PM_2.5_

We used a data source derived from satellite aerosol remote sensing data products such as the Moderate Resolution Imaging Spectroradiometer (MODIS) Collection six level 2 aerosol products at 10 km resolution from Aqua and Terra satellites (http://ladsweb.nascom.nasa.gov/) as well as other information. This PM_2.5_ data set was generated by applying machine learning algorithms to predict historical PM_2.5_ concentrations with satellite-retrieved aerosol optical depth from MODIS, gridded meteorological parameters, as well as land use information in China as predictors (Xiao et al., 2018). Compared with ground monitoring data, satellite data with a broad spatial coverage (all of China), long-term records (from March 2000 to December 2015), and high spatial resolution (10 km) support the assessment of historical air pollution levels in developing regions (Xiao et al., 2018). To validate PM_2.5_ estimates from satellite data, the data set creators conducted the 10-fold cross validation (CV) to evaluate the prediction performance, with a CV *R*^2^ of 0.79, which was significantly higher than previous methods (Xiao et al., 2018). Note that although these PM_2.5_ data were detailed in each (10 km × 10 km) grid cell every month starting in March 2000, the CHARLS did not provide the exact residence address for each respondent due to concerns about identifiability. Thus, we aggregated PM_2.5_ concentrations from satellite data at the city level because the city information from the primary sampling units (PSU) of CHARLS is the smallest spatial unit. In the CHARLS, there were 125 cities selected as the PSU, which were distributed across 28 of 34 provinces, covering 95% of the population in China.

In our study, the period of exposure to PM_2.5_ is from March of 2000 to the month preceding cognitive assessment in each wave of the CHARLS. Supplementary Figure S1 shows the period of exposure for the CHARLS samples in each wave. Considering the evidence of the nonlinear association between air pollution and cognitive function (Ailshire & Crimmins, 2014; Power et al., 2011), we analyzed PM_2.5_ exposure as a categorical variable. Following previous studies related to exposure to risk factors (De Vocht et al., 2015; Pope et al., 2011), there were three subindices for exposure to PM_2.5_ in this study.

The first one was the average concentrations of PM_2.5_ as the “PM_2.5_ intensity.” As the National Ambient Air Quality Standard for annual mean PM_2.5_ is 35 μg/m^3^ (level 1) and 75 μg/m^3^ (level 2) in China (Cao et al., 2013) and the median of the average PM_2.5_ concentration during the period between 2000 and 2015 was 50 μg/m^3^, we categorized PM_2.5_ intensity into four groups using the following cut-points: 1 (0–35 μg/m^3^), 2 (36–50 μg/m^3^), 3 (51–75 μg/m^3^), and 4 (76+ μg/m^3^). The second measure was the duration of exposure, measured as months over a fixed PM_2.5_ concentration threshold. Evidence on the effects of PM_2.5_ in China suggests that exposure to concentration over 50 μg/m^3^ has an adverse impact on cognitive function (Wang et al., 2020). Thus, this study used 50 μg/m^3^ (which is also the median of PM_2.5_ intensity) as the threshold to establish the duration of exposure. Following previous studies (Ranft et al., 2009; Tallon et al., 2017; Zeng et al., 2010), “PM_2.5_ duration” was categorized into four groups: 1 (0–12 months), 2 (13–60 months), 3 (61–120 months), and 4 (121+ months). For the third measure, “cumulative exposure,” we interacted intensity with duration, establishing a joint variable with 16 categories as the cumulative PM_2.5_ indicator. This takes into consideration that regions with higher levels of pollution intensity were likely to have had those higher levels for longer durations, which would have caused a problem of multicollinearity if assessed simultaneously in the same model (details shown in Supplementary Table S11).

### Covariates: Demographic, Socioeconomic Status (SES), and Regional Factors

In this study, we categorized covariates into three groups. The first is demographic information, including sex, age, and partnership status. Women may have a higher risk of cognitive decline associated with increased PM_2.5_ exposure than men (Kim et al., 2019). To account for the well-established curvilinear association between cognitive function and age (Ballesteros et al., 2009), we included both age and a quadratic function for age in our analysis. Living with a partner might have a protective effect against cognitive impairment, especially in later life (Håkansson et al., 2009), so we include a time variant control for partnership status: single (separate, divorced, widowed, or never married) or partnered (married or living with a partner).

The second set of covariates measures socioeconomic status (SES): education at baseline, primary occupational attainment (time-invariant), and time-variant household expenditure. Educational attainment was the most substantial predictor of cognitive function in China as elsewhere (Cagney & Lauderdale, 2002; Huang & Zhou, 2013). Occupational attainment is also associated with cognition. For example, civil servants and managers have a better cognitive function, net of potential confounders (Myung et al., 2016). In this study, occupational attainment was measured by the main job reported during respondents’ occupational history and includes three categories: agricultural, nonagricultural, and managerial occupations. Wealth has been found to be associated with lower cognitive function, so we controlled household expenditure (logged) as an operationalization of wealth (Cagney & Lauderdale, 2002).

HuKou is a household registration system in China that has two categories: rural and urban. People usually remain as the same HuKou as their parents, as once HuKou is registered, it is difficult to change even if people move (Hou et al., 2019). Particularly before China enacted the reform and opening-up policy in 1978, HuKou stipulated whether people could work in industrial sectors, have more educational opportunities, and even have better access to medical insurance (Walder, 1995). As such, HuKou is strongly related to individual SES in addition to residential locations (Hou et al., 2019).

Rapid urbanization and industrialization are associated with improvements in population health (e.g., improved health care system and more health facilities), alongside high levels of air pollution (Gong et al., 2012). In this study, we included annual regional gross domestic product (GDP) at the city level (logged) to reflect the urbanization and industrialization of cities to adjust for this potential confounding factor (Sun & Gu, 2008).

### Analysis Strategies

This study analyzed whether changes in individual cognitive function during the CHARLS 2011–2015 are related to cumulative PM_2.5_ exposure. We employed GCM to examine the relationship between exposure to PM_2.5_ between 2000–2015 and the trajectory of cognitive decline between 2011–2015. An important advantage of GCM is the ability to model the trajectories of individuals over time and distinguish within-individual from between-individual heterogeneity in estimating cognitive changes shaped by other variables. GCM is a special case of random-coefficient models that can take a variety of shapes of growth trajectories, and it is the time coefficient (here, age) that varies randomly between participants (Rabe-Hesketh & Skrondal, 2012). In this study, we used three waves of longitudinal data across 4 years of data collection.

We estimated three separate sets of models, each with different operationalisations of air pollution exposure. The first set of models includes intensity, as the average concentration of PM_2.5_ during the period of exposure. The second set includes duration measured as the number of months where PM_2.5_ concentration is over the threshold of 50 μg/m^3^ during the period of exposure. We tested different thresholds for PM_2.5_ duration as robustness checks (Supplementary Material). The third set includes a joint variable of intensity with duration. To investigate the rate of cognitive decline, we used the interaction term between age, age squared, and PM_2.5_ exposure to examine the association between PM_2.5_ exposure and cognitive trajectories. This is to determine whether higher exposure leads to a faster rate of cognitive decline or just overall lower cognitive function. To examine heterogeneity in the associations of PM_2.5_ exposure among different groups, we also stratified the associations between PM_2.5_ exposure and cognitive function by HuKou status.

We conducted various robustness checks. First, we ran the same models using the imputed data (through MI) to check consistency and verify that our results were not biased by missing data (shown in Supplementary Tables S3–S5). Second, we set up alternative specifications to check the sensitivity of the threshold value in PM_2.5_ exposure variable. For PM_2.5_ intensity, there were six categories using 10 μg/m^3^ as the interval from 35 to 75 μg/m^3^; for PM_2.5_ duration, taking 45 μg/m^3^ as another threshold, and then the joint intensity-duration variable expanded to 24 categories. All of these analyses can be found in Supplementary Tables S6–S8. Third, although the main analysis with PM_2.5_ categories establishes the curvilinear relationship between PM_2.5_ exposure and cognitive function trajectories, categorizing PM_2.5_ records sacrifices some details compared with using continuous PM_2.5_ concentrations. Therefore, we also estimated a model with a quadratic term of continuous exposure (in both intensity and duration) to examine the curvilinear dose–response curve (Supplementary Tables S9 and S10). Fourth, we have added a robustness check using the data that includes only individuals who participated in all three waves of CHARLS (there are 6,589 respondents, including 19,767 observations). This ensures all respondents have the same possibility of exposure ranging from 0 to 328 months (the total observation period) over PM_2.5_ intensity of 50 μg/m^3^ (Supplementary Figure S6).

## Results

Our study population includes 12,481 respondents (29,484 observations) from three waves of the CHARLS 2011, 2013, and 2015. The average age of 2011 entrants was 59 years old, with some variations by PM_2.5_ exposure groups; 49% were men and 76% of the population attained primary or higher education (Table 1). 76% of the study population had rural HuKou, 72% of the population worked in agricultural jobs, and 88% were partnered. The average cognitive function score was 10. Respondents with higher education (e.g., primary or higher), higher household expenditure (8.30), and in areas with higher GDP (10.55) had higher exposure to PM_2.5_ (especially exposed to PM_2.5_ over 76 μg/m^3^).

**Table 1.** Characteristics of 2011 Entrants in the CHARLS by PM_2.5_ Intensity

	Total	PM_2.5_ (μg/m^3^) mean average monthly exposure between 2000 and baseline (mean [*SD*] or *n* [%])			
		1 (0–35 μg/m^3^)	2 (36–50 μg/m^3^)	3 (51–75 μg/m^3^)	4 (76+ μg/m^3^)
Cognitive score	9.97 (4.48)	9.75 (4.44)	9.88 (4.52)	9.92 (4.56)	10.10 (4.39)
Age	58.99 (9.47)	58.14 (9.49)	59.09 (9.48)	59.46 (9.58)	57.55 (8.43)
Gender (%)					
Men	6,069 (48.63)	955 (48.22)	1,697 (48.22)	2,918 (49.02)	499 (47.34)
Women	6,421 (51.37)	1,000 (51.15)	1,822 (51.78)	3,035 (50.98)	555 (52.66)
Education (%)					
No schooling	3,199 (25.63)	642 (32.84)	860 (24.44)	1,537 (25.82)	160 (15.18)
Primary	5,011 (40.15)	808 (41.33)	1,388 (39.44)	2,453 (41.21)	362 (34.35)
Middle	4,271 (34.22)	505 (25.83)	1,271 (36.12)	1,963 (32.97)	532 (50.47)
HuKou (%)					
Rural	9,705 (77.76)	1,694 (86.61)	2,596 (73.77)	4,582 (76.97)	833 (79.03)
Urban	2,776 (22.24)	261 (13.39)	923 (26.23)	1,371 (23.03)	221 (20.97)
Occupation (%)					
Agricultural	9,038 (72.41)	1,506 (77.03)	2,389 (67.89)	4,405 (74.00)	738 (70.02)
Nonagricultural	2,789 (22.35)	358 (18.31)	923 (26.23)	1,263 (21.22)	245 (23.24)
Managerial	654 (5.24)	91 (4.65)	207 (5.88)	285 (4.79)	71 (6.74)
Partnership status (%)					
Partnered	11,014 (88.25)	1,684 (86.14)	3,054 (86.79)	5,330 (89.53)	946 (89.75)
Single	1,467 (11.75)	271 (13.86)	465 (13.21)	623 (10.47)	108 (10.25)
Log household expenditure	8.16 (1.67)	8.18 (1.66)	8.15 (1.82)	8.14 (1.57)	8.30 (1.74)
Log GDP	10.29 (0.55)	10.19 (0.51)	10.21 (0.63)	10.33 (0.50)	10.55 (0.43)
Number of respondents (%)	12,481	1,955 (15.66)	3,519 (28.19)	5,953 (47.71)	1,054 (8.44)

*Notes*: All statistics are calculated after list wise deletion (see details in the “Method” section). The characteristics of a cognitive score, age, log household expenditure and log GDP are shown using mean (*SD*), and others are in *N* (%). CHARLS = China Health and Retirement Longitudinal Study; *SD* = standard deviation; GDP = gross domestic product.

Table 2 shows the results from the GCMs. Model 1 is the base model with PM_2.5_ intensity, age, age squared, and gender. The coefficients in Model 1 show that the associations between PM_2.5_ intensity and cognitive function are positive (higher PM_2.5_ is associated with better cognitive function). However, after education was included in Model 2 as a confounding variable, the association between PM_2.5_ intensity and cognitive function takes a U-shaped pattern, with both lower and higher intensity being associated with higher cognitive function, a finding to which we return below. In Model 3, which controls additionally for occupation, household expenditure, and partnership status, we found that compared with people exposed to the lowest PM_2.5_ intensity (0–35 μg/m^3^), those in the next group of higher exposure (36–50 μg/m^3^) had lower cognitive function (β = −0.252, *p* < .01); however, the fourth group (those with the highest levels of exposure) had a better cognitive function—an unexpected result. This could be caused by SES confounding (details in marginal plots in Supplementary Figure S2). Model 4 (including logged GDP), indeed, supports the U-shaped results of Models 2 and 3.

**Table 2.** Associations Between PM_2.5_ Intensity and Cognitive Function

	Model 1: Base	Model 2: Model 1 + Education	Model 3: Model 2 + SES + Partnership	Model 4: Model 3 + GDP
PM_2.5_ intensity (ref: 0–35 μg/m^3^)				
2 (36–50)	0.374***	−0.141	−0.252**	−0.233**
	(0.173 to 0.575)	(−0.320 to 0.0373)	(−0.428 to −0.0769)	(−0.408 to −0.0577)
3 (51–75)	0.500***	0.0363	−0.0291	−0.0443
	(0.314 to 0.685)	(−0.128 to 0.200)	(−0.190 to 0.132)	(−0.205 to 0.116)
4 (76+)	1.087***	0.199#	0.197#	0.118
	(0.835 to 1.339)	(−0.0271 to 0.425)	(−0.0248 to 0.419)	(−0.105 to 0.340)
Age	0.127***	0.249***	0.236***	0.234***
	(0.0560 to 0.197)	(0.185 to 0.313)	(0.172 to 0.299)	(0.171 to 0.297)
Age squared	−0.00231***	−0.00275***	−0.00269***	−0.00269***
	(−0.0029 to −0.0018)	(−0.0033 to −0.0023)	(−0.0032 to −0.0022)	(−0.0032 to −0.0022)
Gender (ref: men)				
Women	−1.286***	−0.0665	−0.116#	−0.143*
	(−1.415 to −1.157)	(−0.187 to 0.0540)	(−0.235 to 0.00329)	(−0.262 to −0.0235)
Education (ref: no-schooling)				
Primary		2.626***	2.414***	2.356***
		(2.476 to 2.776)	(2.266 to 2.563)	(2.207 to 2.505)
Middle		5.053***	4.320***	4.247***
		(4.887 to 5.219)	(4.144 to 4.496)	(4.071 to 4.424)
HuKou (ref: rural)				
Urban			1.165***	1.130***
			(1.010 to 1.320)	(0.976 to 1.285)
Occupation (ref: agricultural)				
Nonagricultural			0.475***	0.440***
			(0.327 to 0.623)	(0.292 to 0.588)
Managerial			0.294*	0.273*
			(0.0619 to 0.525)	(0.0410 to 0.504)
Log household expenditure			0.0912***	0.0876***
			(0.0668 to 0.116)	(0.0632 to 0.112)
Partnership (ref: partnered)				
Single			−0.349***	−0.340***
			(−0.516 to −0.183)	(−0.507 to −0.174)
Log GDP				0.332***
				(0.239 to 0.426)
Constant	10.98***	2.265*	2.180*	−1.060
	(8.785 to 13.17)	(0.262 to 4.267)	(0.181 to 4.179)	(−3.254 to 1.135)
Random effects				
Within individual				
Change rate (age)	0.003***	0.005***	0.004***	0.004***
Intercept	2.931***	2.797***	2.660***	2.663***
Covariance	0.014	−0.008	−0.008	−0.009
Between individual				
Residuals	3.290***	3.051***	3.056***	3.056***
Log likelihood	−82,019.908	−80,455.387	−80,218.955	−80,194.873
Observations	29,484	29,484	29,484	29,484
Number of IDs	12,481	12,481	12,481	12,481

*Notes*: Cognitive function includes three components: immediate recall, delayed recall, and serial 7s, 0–25 points (see details in “Methods”). SES = socioeconomic status; GDP = gross domestic product.

***p < .001, **p < .01, *p < .05, #*p* < .1.

The associations between cognitive function and other covariates in Model 4 were as expected. First, women have poorer cognitive function than men. Age and age squared show the expected curvilinear association with cognitive function. The log-likelihood test for age and age squared in Supplementary Table S14 also shows the necessity of the quadratic age term. Higher SES across all three indicators (urban HuKou, nonagricultural occupation, and higher household expenditure) is associated with higher cognitive function. Unpartnered individuals have lower cognitive scores compared with their partnered counterparts. Model 4 also shows a positive association between GDP and cognitive function.

In Model 4 (of Table 2), the random intercept standard deviation is 2.7 (*p* < .001), reflecting the significant variation in average cognitive function scores between individuals. The small standard deviation for the age random slope (0.003, *p* < .01) reflects the fact that cognitive decline follows a relatively predictable, downward trajectory with age. Similar findings were shown in previous studies, including ones that assessed other important factors that might predict the rate of cognitive decline, such as race/ethnicity (Hale, 2017), education (Seblova et al., 2020), and neighborhood environment (Luo et al., 2019).

Table 3 presents results from the models that analyze the duration of exposure. Model 1 shows positive associations between PM_2.5_ duration and cognitive function score, but after controlling for education, increased duration of exposure to higher PM_2.5_ was negatively associated with cognitive function only for those in the second group of PM_2.5_ duration (13–60 months). The association was nonsignificant for the two longer-duration groups (61–120 and 121+ months). In Model 3, SES variables explained a part, but not all, of the associations. In Model 4, when all covariates were included, the associations of PM_2.5_ duration were similar to Models 2 and 3, suggesting people in the second group of exposure duration were more likely to have the poorer cognitive function, while the third and fourth groups were not statistically different than the first. The strong association for duration group 2 compared with the other groups is addressed at length later.

**Table 3.** Associations Between PM_2.5_ Duration (at a Threshold of 50 μg/m^3^) and Cognitive Function

	Model 1: Base	Model 2: Model 1 + Education	Model 3: Model 2 + SES + Partnership	Model 4: Model 3 + GDP
PM_2.5_ duration (ref: 1 [0–12 months])				
2 (5 years: 13–60 months)	0.0319	−0.437***	−0.541***	−0.495***
	(−0.150 to 0.214)	(−0.604 to −0.270)	(−0.706 to −0.377)	(−0.661 to −0.330)
3 (10 years: 61–120 months)	0.306***	−0.0785	−0.128#	−0.125#
	(0.139 to 0.472)	(−0.229 to 0.0715)	(−0.275 to 0.0198)	(−0.272 to 0.0228)
4 (10+ years: 121 months+)	0.509***	−0.0734	−0.0981	−0.155#
	(0.329 to 0.689)	(−0.236 to 0.0895)	(−0.258 to 0.0622)	(−0.316 to 0.00597)
Age	0.125***	0.252***	0.239***	0.238***
	(0.0542 to 0.196)	(0.188 to 0.316)	(0.175 to 0.302)	(0.175 to 0.301)
Age square	−0.00231***	−0.00277***	−0.00270***	−0.00271***
	(−0.0029 to −0.0018)	(−0.0033 to −0.0023)	(−0.0032 to −0.0022)	(−0.0032 to −0.0022)
Gender (ref: men)				
Women	−1.286***	−0.0564	−0.105#	−0.131*
	(−1.415 to −1.157)	(−0.177 to 0.0641)	(−0.224 to 0.0138)	(−0.250 to −0.0123)
Education (ref: no-schooling)				
Primary		2.642***	2.430***	2.372***
		(2.492 to 2.792)	(2.281 to 2.578)	(2.223 to 2.520)
Middle		5.095***	4.359***	4.287***
		(4.929 to 5.260)	(4.183 to 4.535)	(4.111 to 4.464)
HuKou (ref: rural)				
Urban			1.178***	1.143***
			(1.023 to 1.332)	(0.988 to 1.298)
Occupation (ref: agricultural)				
Nonagricultural			0.475***	0.440***
			(0.326 to 0.623)	(0.291 to 0.588)
Managerial			0.313**	0.291*
			(0.0819 to 0.545)	(0.0597 to 0.523)
Log household expenditure			0.0907***	0.0873***
			(0.0664 to 0.115)	(0.0629 to 0.112)
Partnership (ref: partnered)				
Single			−0.349***	−0.342***
			(−0.516 to −0.183)	(−0.508 to −0.176)
Log GDP				0.324***
				(0.229 to 0.418)
Constant	11.27***	2.264*	2.165*	−1.035
	(9.075 to 13.47)	(0.260 to 4.268)	(0.166 to 4.165)	(−3.240 to 1.170)
Random effects				
Within individual				
Change rate (age)	0.002***	0.005***	0.004***	0.004***
Intercept	2.946***	2.801***	2.663***	2.664***
Covariance	0.139	−0.008	−0.008	−0.009
Between individual				
Residuals	3.053***	3.049***	3.054***	3.054***
Log lokelihood	−82,035.322	−80,444.162	−80,204.329	−80,181.936
Observations	29,484	29,484	29,484	29,484
Number of IDs	12,481	12,481	12,481	12,481

*Notes*: Cognitive function includes three components: immediate recall, delayed recall, and serial 7s, 0–25 points (see details in “Methods”). SES = socioeconomic status; GDP = gross domestic product.

***p < .001, **p < .01, *p < .05, #*p* < .1.

As well as considering PM_2.5_ intensity and duration separately, we were also interested in their joint or cumulative association with cognitive function. At baseline, the individual distribution of cumulative PM_2.5_ exposure (intensity × duration) from March of 2000 to the survey date was shown in Supplementary Table S11. Due to small numbers in the “3–2” group (only 169), we merged that group into the “3–3” group. Table 4 shows the associations between cumulative exposure (measured by a joint variable of intensity with duration) and cognitive function. In Model 1, compared with the “1–1” group (lowest intensity and shortest duration), higher levels of intensity-duration were associated with better cognitive function, except for the group of “1–2,” which suggests people in that group had lower cognitive function than those in the “1–1” group. In Model 2, when education was included, most coefficients of PM_2.5_ exposure were reversed to negative, suggesting that education was a confounder of the association between PM_2.5_ exposure and cognitive function. When more SES covariates and partnership status were added in Model 3, the negative patterns between cumulative exposure to PM_2.5_ and cognitive function persisted for most groups, though the most groups were nonsignificant. These results were also robust in Model 4 when logged GDP was controlled. We found the effect size of the “1–2” group was significantly larger than other groups, which is worth exploring in future analyses. Comparing the coefficients of “1–2” (longer duration) and “2–1” (higher intensity) group suggests that duration might be more significantly associated with cognitive function than intensity.

**Table 4.** Associations Between Cumulative PM_2.5_ Exposure (Intensity-Duration) and Cognitive Function

	Model 1: Base	Model 2: Model 1 + Education	Model 3: Model 2 + SES + Partnership	Model 4: Model 3 + GDP
Cumulative PM_2.5_ (ref: 1–1)				
1–2	−0.612*	−0.775***	−0.691**	−0.684**
	(−1.098 to −0.125)	(−1.214 to −0.337)	(−1.123 to −0.260)	(−1.115 to −0.253)
2–1	0.394**	0.125	0.102	0.0850
	(0.0969 to 0.691)	(−0.144 to 0.393)	(−0.163 to 0.366)	(−0.179 to 0.349)
2–2	0.202#	−0.399***	−0.523***	−0.482***
	(−0.0192 to 0.423)	(−0.597 to −0.202)	(−0.718 to −0.329)	(−0.676 to −0.287)
2–3	0.522***	−0.0327	−0.146	−0.156
	(0.246 to 0.797)	(−0.287 to 0.222)	(−0.398 to 0.105)	(−0.408 to 0.0950)
3–3	0.419***	−0.0292	−0.0886	−0.0869
	(0.222 to 0.617)	(−0.205 to 0.146)	(−0.261 to 0.0836)	(−0.259 to 0.0849)
3–4	0.486***	−0.0895	−0.143	−0.195*
	(0.269 to 0.702)	(−0.285 to 0.106)	(−0.335 to 0.0487)	(−0.388 to −0.00315)
4–4	1.027***	0.107	0.115	0.0359
	(0.768 to 1.285)	(−0.124 to 0.339)	(−0.112 to 0.342)	(−0.192 to 0.264)
Age	0.124***	0.251***	0.239***	0.238***
	(0.0527 to 0.194)	(0.187 to 0.315)	(0.175 to 0.302)	(0.175 to 0.302)
Age squared	−0.00229***	−0.00276***	−0.00270***	−0.00271***
	(−0.0029 to −0.0017)	(−0.0033 to −0.0023)	(−0.0032 to −0.0022)	(−0.0032 to −0.0022)
Gender (ref: men)				
Women	−1.288***	−0.0608	−0.109#	−0.134*
	(−1.417 to −1.159)	(−0.181 to 0.0597)	(−0.228 to 0.0101)	(−0.253 to −0.0154)
Education (ref: no-schooling)				
Primary		2.635***	2.423***	2.366***
		(2.485 to 2.785)	(2.275 to 2.571)	(2.217 to 2.515)
Middle		5.078***	4.342***	4.273***
		(4.912 to 5.244)	(4.166 to 4.518)	(4.096 to 4.450)
HuKou (ref: rural)				
Urban			1.177***	1.143***
			(1.023 to 1.332)	(0.988 to 1.297)
Occupation (ref: agricultural)				
Nonagricultural			0.477***	0.442***
			(0.329 to 0.625)	(0.294 to 0.590)
Managerial			0.313**	0.291*
			(0.0810 to 0.544)	(0.0593 to 0.522)
Log household expenditure			0.0901***	0.0866***
			(0.0657 to 0.114)	(0.0623 to 0.111)
Partnership (ref: partnered)				
Single			−0.350***	−0.343***
			(−0.517 to −0.184)	(−0.509 to −0.176)
Log GDP				0.319***
				(0.225 to 0.414)
Constant	11.15***	2.238*	2.132*	−1.022
	(8.949 to 13.35)	(0.231 to 4.245)	(0.129 to 4.136)	(−3.230 to 1.186)
Random effects				
Between individual				
Change rate (age)	0.003***	0.005***	0.004***	0.004***
Intercept	2.930***	2.792***	2.653***	2.655***
Covariance	0.007***	−0.009	−0.010	−0.010
Within individual				
Residuals	3.052***	3.048***	3.054***	3.054***
Log likelihood	−82,012.150	−80,437.023	−80,197.855	−80,176.084
Observations	29,484	29,484	29,484	29,484
Number of IDs	12,481	12,481	12,481	12,481

*Notes*: Cognitive function includes three components: immediate recall, delayed recall, and serial 7s, 0–25 points (see details in “Methods”). The first number in the cumulative PM_2.5_ is intensity (1: 0–35 μg/m^3^; 2: 36–50 μg/m^3^; 3: 51–75 μg/m^3^; 4: 76+ μg/m^3^), and the second represents duration (1: 0–12 months; 2: 13–60 months; 3: 61–120 months; 4: 121+ months). SES = socioeconomic status; GDP = gross domestic product.

***p < .001, **p < .01, *p < .05, #*p* < .1.

**Figure 2.**
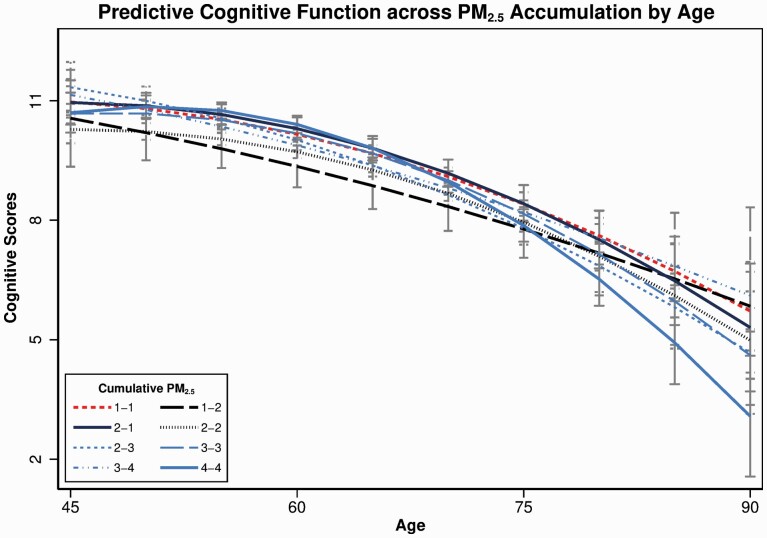
Trajectories of cognitive function associated with cumulative PM_2.5_ (intensity-duration) across age with 95% confidence intervals. Adjusted covariates were from Model 5 in Supplementary Table S13. The first number in the cumulative PM_2.5_ is intensity (1: 0–35 μg/m^3^; 2: 36–50 μg/m^3^; 3: 51–75 μg/m^3^; 4: 76+ μg/m^3^), and the second represents duration (1: 0–12 months; 2: 13–60 months; 3: 61–120 months; 4: 121+ months).

To test whether PM_2.5_ exposure is associated with accelerated cognitive decline, based on Model 4 in Table 4, Model 5 adds interaction terms between cumulative exposure, age, and age squared (full results in Supplementary Table S13). Figure 2 plots predicted cognitive function by cumulative PM_2.5_ across age, visually depicting that level of cumulative PM_2.5_ exposure is not statistically significantly associated with different trajectories (details in Supplementary Table S13). We found no statistically significant associations between higher exposure for longer duration groups (e.g., “3–3” or “4–4” groups) and faster rate of cognitive decline.

**Figure 3.**
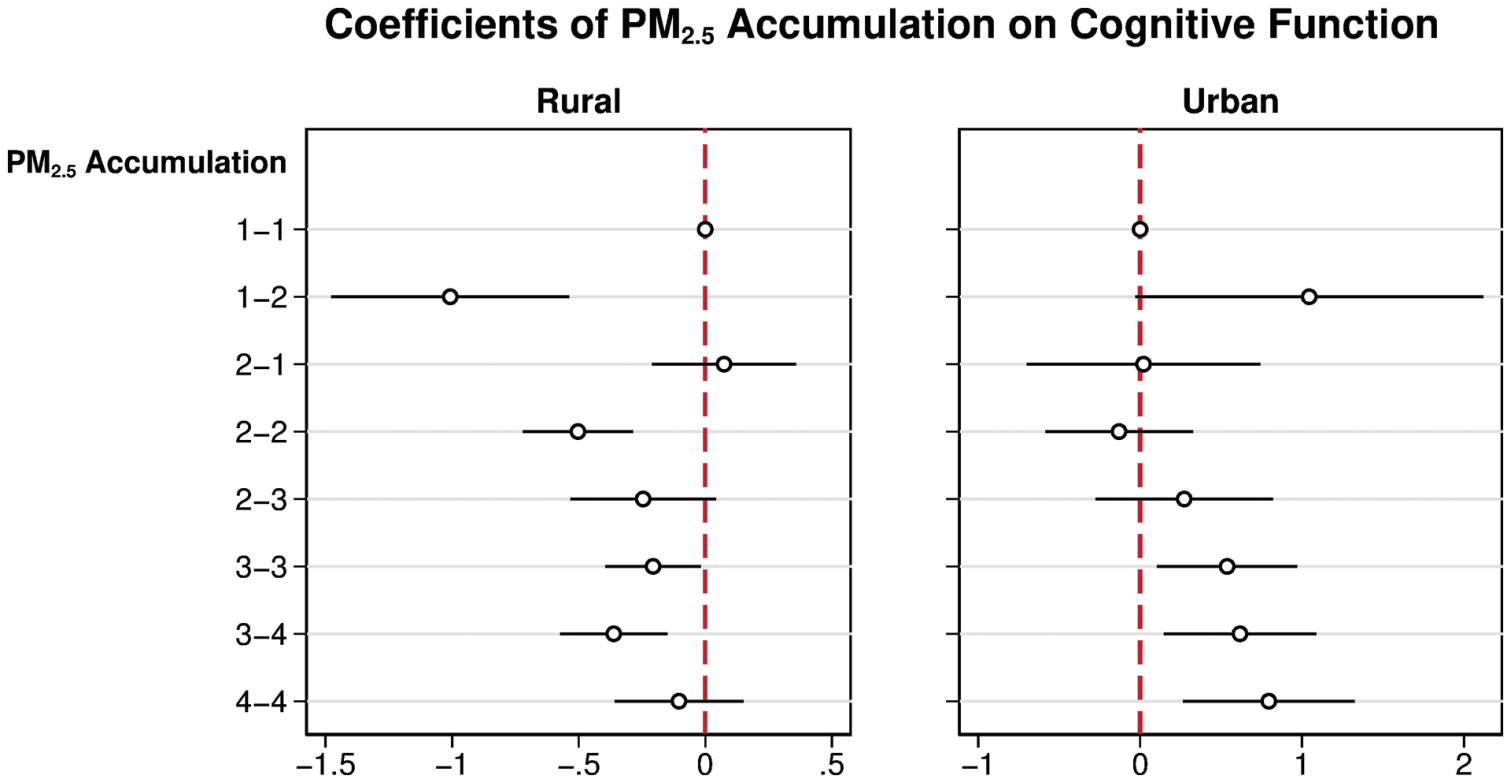
Associations between cumulative PM_2.5_ exposure (intensity-duration) and cognitive function stratified by HuKou status. Adjusted covariates include age, age squared, gender, education, occupation, household expenditure (logged), and annual GDP at the city level (logged). GDP = gross domestic product.

Considering the significant difference in education and air pollution between urban and rural areas, we also stratified the analysis by HuKou status. Figure 3 displays the coefficients for PM_2.5_, which suggest that the harmful associations of cumulative air pollution exposure were more significant among respondents with rural HuKou than those with urban HuKou. Supplementary Figures S4 and S5 show the coefficients of PM_2.5_ intensity and duration.

We also conducted robustness checks. First, we ran all of the above models using the MI data sets (see Supplementary Tables S3–S5). Comparing all results in Tables 2–4 with Supplementary Tables S3–S5, we found that there were no meaningful differences. Second, when we divided PM_2.5_ intensity into six categories (Supplementary Table S6) for the data with MI, the findings were similar to Table 2, but with reduced effect size and not always significant. Results for the duration and the cumulative measure (Supplementary Tables S7 and S8) were also consistent with the main findings (Tables 3 and 4), suggesting an association of more intensive cumulative PM_2.5_ exposure with lower cognitive function scores. Third, Supplementary Tables S9 and S10 (using continuous variable for PM_2.5_ exposure) suggest that this association between PM_2.5_ exposure and cognitive function may not be a quadratic curve but a more complex curvilinear relationship. Fourth, the robustness check using the fully balanced data, with a smaller sample size (e.g., no attritors; Supplementary Figure S6) confirms that cumulative exposure remains associated with poor cognitive function, but these associations diminish for PM_2.5_ exposures of high intensity and high duration. This is possibly because those who attritted are more likely to have poor health.

## Discussion

Using the CHARLS, a nationally representative data set, linked to historic PM_2.5_ records derived from remotely sensed satellite data, we investigated the relationship between PM_2.5_ exposure at the city level and cognitive function via a sequence of GCMs. Our findings were mixed but suggested that some categories of higher and longer exposure to PM_2.5_ are associated with poorer cognitive function among Chinese adults aged 45 and older.

First, we used the intensity (average PM_2.5_ exposure) as the measure for exposure over a long-term period of 15 years. Our findings show that respondents in the second group of PM_2.5_ intensity have poorer cognitive function than those in the first group, but their associations retained only weak levels of significance once adjusted for education and other SES factors. Moreover, our findings in Table 2 and Supplementary Table S3 show that people living in areas of much higher PM_2.5_ intensity (e.g., categories three and four) have a better cognitive function.

Although some studies show no significant associations between PM_2.5_ intensity and cognitive impairment (Gatto et al., 2014; Loop et al., 2013), our findings are consistent with most of the previous research, which indicates higher mean concentrations of PM_2.5_ were significantly associated with lower cognitive function (Ailshire & Clarke, 2015; Ailshire et al., 2017; Tallon et al., 2017; Wang et al., 2020). We have controlled for many individual and city-level confounders, but as in most observational studies, we cannot rule out residual confounding factors related to air pollution or cognitive function, or both, which might result in high between-person residuals and unexpected patterns of associations between air pollution and cognitive function.

Although our study adjusts for HuKou status, this may not capture every important element of rural–urban differences. For example, economic development and urbanization correlate with high pollution and high rates of rural–urban migration (Chai et al., 2014; Liu et al., 2007; Zhang & Kanbur, 2005). Therefore, the negative association between higher air pollution (e.g., the highest category of PM_2.5_ intensity or duration) and poorer cognition might be offset by the advantageous characteristics of urban dwellers. This might explain why the analyses stratified by HuKou show that rural respondents had stronger associations between PM_2.5_ exposure and poor cognitive function than urban ones. We additionally included measures of educational attainment and other SES covariates (household expenditure and occupation), but these also may not capture all rural–urban differences (e.g., medical insurance, housing, and community services). Furthermore, the remaining high between-person residuals might be due to measuring air pollution exposure at the city-level resolution. In China, the city region may consist of rural and urban areas where air pollution exposures are remarkably different. This should be explored in future research with the different data source(s) that allow the inclusion of more potential confounding factors and more precise spatial resolution.

Second, our results suggest that exposure to 13–60 months of PM_2.5_ duration was associated with worse cognitive function than the first-group duration (0–12 months), consistent with previous studies regarding the effects of exposure durations (Liu et al., 2017; Tan et al., 2018). However, coefficients for durations longer than 60 months were nonsignificant, and likely for similar reasons as observed with intensity: the stronger association between the second duration group and cognitive function could be caused by some unobserved individual factors (e.g., people living in the regions of the second duration group might have less protection against air pollution) or urbanization factors. Compared with the effect sizes and significance of PM_2.5_ intensity, our findings reflect that PM_2.5_ duration might have more detrimental influences on cognitive function.

Third, the results using a joint measure of intensity with duration for cumulative exposure indicate that the exposure to a longer duration at a lower intensity may be more harmful than a shorter duration at a higher intensity. This suggests that duration of exposure may play a more important role in cognitive impairment than the intensity of exposure, especially in a 15-year period or longer. On the other hand, there might be an alternative explanation for the lower cognitive level of the “1–2” group. After further investigation, we found that more than 80% of the individuals in the “1–2” group were from one particular city (Maoming of Guangdong Province), where urbanization dramatically increased the level of PM_2.5_ concentration during the last 15 years (Maoming Municipal Government, 2020). Supplementary Figure S3 shows that there were only 16 months with more than 50 μg/m^3^ at PM_2.5_ concentration, which indicates most samples in Maoming are exposed to the “1–1” and “1–2” groups. This also partly explains why the coefficients of the third and fourth groups of duration were nonsignificant in Table 4. Therefore, these findings suggest that the measure of air pollution should include intensity and duration simultaneously.

It should be noted that our findings related to duration were substantiated only when we defined that the harmful threshold is more than 50 μg/m^3^ at PM_2.5_ concentration. Evidence shows that poor cognitive performance is associated with a lower level of PM_2.5_ concentration (e.g., 15 μg/m^3^) among American older adults (Ailshire & Clarke, 2015). In China, although the national standard for annual PM_2.5_ concentration is 35 μg/m^3^ (level 1) and 75 μg/m^3^ (level 2; Cao et al., 2013), we found that 85% of respondents in the CHARLS were living in cities where the annual level of PM_2.5_ exposure is over 35 μg/m^3^. Thus, if we used a lower threshold to measure the duration, the variation of duration would be too small to accurately reflect the relationship between PM_2.5_ exposure and cognitive function. In addition, China was experiencing rapid urbanization, which to some extent leads to a positive correlation between human health and air pollution because the increased GDP and improved infrastructure are likely to be the main drivers of beneficial health outcomes; this might have downwardly biased our estimates of the association between air pollution and cognitive function (Hou et al., 2019).

Several limitations should be noted. First, because we did not have access to respondents’ address data, we matched individuals to PM_2.5_ exposure data based on the city where they lived; hence, we cannot compare respondents within the same city. Future studies that can match air pollution data at the individual level (not possible with our data) could produce more robust results. This study can only indicate the associations between PM_2.5_ exposure and cognitive function, but cannot examine the effects of other air pollutants, such as NO_2_, PM_10_, and ozone, which are also associated with cognitive impairment (Kulick et al., 2020; Shin et al., 2018). Thus, we cannot rule out that our findings might result from other pollutants that are highly correlated with PM_2.5_. Finally, although we use long-term PM_2.5_ exposure data measured over a 15-year period, we have no measure of the earlier life course exposure.

Nevertheless, our study makes two important contributions. First, we extend the measure of air pollution exposure beyond exposure intensity (average PM_2.5_ concentrations) to exposure duration (the number of months with high levels of PM_2.5_ concentrations) and cumulative exposure (a joint variable of intensity with duration), providing a more comprehensive assessment of the associations between cumulative PM_2.5_ exposure and cognitive function. Second, compared with monitoring station data in China, the satellite-based PM_2.5_ data used in this study have many methodological advantages, including a broad spatial coverage (all of China) and long-term records (more than 15 years). Third, the linkage between survey data and satellite data is established using exact interview dates and locations over a period of 15 years, enabling us to accurately observe the temporal trends of PM_2.5_ exposure, even if interview dates varied between respondents. Using a long-term exposure period is important when studying cognitive impairment, because the initial deposition of brain diseases (e.g., dementia and Alzheimer’s disease) may begin at least 10–15 years before clinically detectable symptoms associated with cognitive impairment (Fortea et al., 2021; Tarawneh & Holtzman, 2012).

## Conclusions

This study contributes to the accumulating literature linking cumulative exposure to PM_2.5_ and cognitive function. We find some evidence that higher and longer PM_2.5_ exposure is associated with worse cognitive function. This suggests that studies of the association between PM_2.5_ exposure and cognitive function should consider both intensity and duration simultaneously. On the other hand, we consistently find that those with the lowest exposure to PM_2.5_ have the lowest levels of cognitive function. The relationship is likely complicated by the association between SES, residence, and migration patterns. Future studies should try to unpick the influence of these factors, and to better understand the causal mechanisms underlying the association between cumulative exposure to PM_2.5_ and cognitive decline.

## Supplementary Material

gbac162_suppl_Supplementary_Material
