# Supplementary material for: A Longitudinal Analysis of the Association Between Long-Term Exposure to Air Pollution and Cognitive Function Among Adults Aged 45 and Older in China
Source: J Gerontol B Psychol Sci Soc Sci. 2022 Oct 10;78(3):556–69. doi: 10.1093/geronb/gbac162 (PMC9985317; doi:10.1093/geronb/gbac162)
Supplement: gbac162_suppl_Supplementary_Material [file gbac162_suppl_supplementary_material.pdf]

Supplemental Material

Imputation process

Due to over 25% of respondents having missing data in CHARLS, we used multiple imputation (MI) to complete our samples using a multivariate, regression-based procedure in Stata 16.0. Following the method of MI with chained equations for HRS (Fisher et al., 2013), we used a combination of relevant demographic, health, and economic variables to perform the imputations.

Our cognitive function measure includes serial 7s, immediate word recall, and delayed recall in our paper (all extracted from the harmonized CHARLS data). We first summarised the three components to generate a variable for cognitive function, and then conducted the MI. Table S1 shows the number of values of missingness for each analysis variable at each wave. Values were imputed to replace missing values (.m), special missing (.p), and refusals (.r).

We imputed all of variables with missingness in this study under the missing at random (MAR) assumption, meaning all variables with missingness can be predicted by the observed data (Carpenter and Kenward, 2013; Rubin, 1976). To avoid bias in the analysis models, the imputation models by chained equations must include all variables that are in the analysis model (White et al., 2011). Given the final analysis models in this study and the suggestions from HRS (Mccammon et al., 2019), our imputation covariates includes time-invariant demographics (gender, education), time-variant demographics (age, marital status), and other time-variant predictors (socioeconomic variables: HuKou, occupations; regional variable: GDP per capita and $PM_{2.5}$ intensity).

Specifically, we use logistic models to impute binary variables (gender and HuKou), ordinal logistic models for educational attainment and occupation, multinomial logistic models for marital status, and ordinary least squared (OLS) for age, household expenditure, and cognitive function. All imputations run 10 times.

After creating multiple imputed data sets, the analysis was rerun once more and the resulting models combined using Rubin's rules (Rubin, 1976), which take into account variation both within and between data sets.

Tables

Table S1. Missing observations from main variables per wave.

	Number of missing observations at each wave		
	2011	2013	2015
Cognitive score	3261	4148	2266
Serial 7s	1711	3640	1369
Immediate	3377	2406	1710
Delayed	3492	2667	2144
Age	20	40	64
Gender	7	5	12
Education	49	33	55
HuKou	25	31	54
Occupation	208	167	134
Marital Status	29	18	40
Log household expenditure	1879	3390	3239
Log GDP	0	0	0

Table S2. Observations before and after multiple imputations.

	N		
	Complete	Incomplete	Imputed
Cognitive score	46674	7430	7430
Age	53977	124	124
Gender	54068	24	24
Education	53967	137	136
HuKou	53987	110	110
Occupation	53595	509	507
Marital Status	54017	87	86
Log household expenditure	45596	8508	8501

Table S3. Associations between $PM_{2.5}$ intensity and cognitive function in multiple imputed data.

	Model 1: Basic	Model 2: Model 1 + Education	Model 3: Model 2 + SES + Marital	Model 4: Model 3 + GDP
$PM_{2.5}$ intensity (ref: 0-35 $\mu g/m^3$)				
2 (36-50)	0.407***	-0.119	-0.228**	-0.209**
	(0.231 - 0.583)	(-0.275 - 0.0369)	(-0.381 - -0.0746)	(-0.362 - -0.0558)
3 (51-75)	0.572***	0.0943	0.0231	0.00569
	(0.408 - 0.736)	(-0.0502 - 0.239)	(-0.119 - 0.165)	(-0.136 - 0.147)
4 (75+)	1.079***	0.213*	0.219*	0.152
	(0.854 - 1.304)	(0.0113 - 0.414)	(0.0219 - 0.416)	(-0.0456 - 0.350)
Age	0.108***	0.235***	0.217***	0.214***
	(0.0463 - 0.169)	(0.179 - 0.290)	(0.162 - 0.272)	(0.159 - 0.269)
Age squared	-0.00225***	-0.00270***	-0.00258***	-0.00258***
	(-0.00273 - -0.0018)	(-0.0031 - -0.0023)	(-0.0030 - -0.0022)	(-0.0030 - -0.0021)
Gender (ref: men)				
Women	-1.361***	-0.118*	-0.167**	-0.190***
	(-1.475 - -1.247)	(-0.225 - -0.0111)	(-0.272 - -0.0606)	(-0.296 - -0.0841)
Education (ref: no-schooling)				
Primary		2.654***	2.428***	2.376***
		(2.521 - 2.786)	(2.298 - 2.559)	(2.245 - 2.507)
Middle		5.102***	4.346***	4.282***
		(4.954 - 5.249)	(4.190 - 4.501)	(4.125 - 4.438)
HuKou (ref: rural)				
Urban			1.226***	1.190***
			(1.090 - 1.362)	(1.054 - 1.327)

Occupation (ref: agricultural)				
Non-Agricultural			0.448***	0.420***
			(0.318 - 0.578)	(0.289 - 0.550)
Managerial			0.349***	0.337**
			(0.142 - 0.556)	(0.130 - 0.544)
Log household expenditure			0.102***	0.0991***
			(0.0797 - 0.125)	(0.0767 - 0.122)
Marital (ref: partnered)				
Single			-0.354***	-0.347***
			(-0.498 - -0.209)	(-0.491 - -0.203)
Log GDP				0.283***
				(0.203 - 0.362)
Constant	11.69***	2.743**	2.676**	-0.0514
	(9.766 - 13.61)	(0.990 - 4.495)	(0.928 - 4.424)	(-1.958 - 1.855)
**Random effects**				
Within individual				
Change rate (Age)	0.013***	0.003***	0.003***	0.003***
Intercept	2.950***	2.675***	2.601***	2.599***
Between individual				
Residuals	3.074***	3.082***	3.084***	3.085***
Observations	39,032	39,032	39,032	39,032
Number of IDs	16,027	16,027	16,027	16,027

Note: Cognitive function includes three components: immediate recall, delayed recall, and serial 7s, 0-25 points (see details in "Methods").
*** p<0.001, ** p<0.01, * p<0.05, # p<0.1

Table S4. Associations between $PM_{2.5}$ duration (at a threshold of 50 $\mu g/m^3$) and cognitive function in multiple imputed data.

	Model 1: Basic	Model 2: Model 1 + Education	Model 3: Model 2 + SES+ Marital	Model 4: Model 3 + GDP
$PM_{2.5}$ duration (ref: 1(0-12 months))				
2 (5 yrs: 13-60 months)	0.173*	-0.291***	-0.379***	-0.345***
	(0.0136 - 0.332)	(-0.437 - -0.145)	(-0.523 - -0.235)	(-0.489 - -0.201)
3 (10 yrs: 61-120 months)	0.434***	0.0147	-0.0368	-0.0439
	(0.286 - 0.581)	(-0.118 - 0.147)	(-0.167 - 0.0935)	(-0.174 - 0.0862)
4 (10+ yrs: 121 months+)	0.557***	-0.0180	-0.0321	-0.0869
	(0.399 - 0.715)	(-0.161 - 0.125)	(-0.172 - 0.108)	(-0.228 - 0.0541)
Age	0.106***	0.237***	0.219***	0.217***
	(0.0446 - 0.168)	(0.182 - 0.293)	(0.164 - 0.274)	(0.163 - 0.272)
Age squared	-0.00224***	-0.00271***	-0.00260***	-0.00260***
	(-0.0027 - -0.0018)	(-0.00315 - -0.0023)	(-0.0030 - -0.0022)	(-0.0030 - -0.0022)
Gender (ref: men)				
Women	-1.360***	-0.112*	-0.160**	-0.183***
	(-1.474 - -1.245)	(-0.219 - -0.00481)	(-0.266 - -0.0539)	(-0.289 - -0.0776)
Education (ref: no-schooling)				
Primary		2.663***	2.438***	2.384***
		(2.531 - 2.796)	(2.307 - 2.568)	(2.253 - 2.516)
Middle		5.129***	4.375***	4.310***
		(4.982 - 5.276)	(4.219 - 4.531)	(4.154 - 4.466)
HuKou (ref: rural)				
Urban			1.226***	1.190***
			(1.090 - 1.362)	(1.054 - 1.326)
Occupation (ref: agricultural)				
Non-Agricultural			0.446***	0.417***
			(0.316 - 0.576)	(0.287 - 0.547)
Managerial			0.361***	0.348***
			(0.154 - 0.568)	(0.141 - 0.555)

Log household expenditure			0.102***	0.0989***
			(0.0793 - 0.124)	(0.0764 - 0.121)
Marital (ref: partnered)				
Single			-0.354***	-0.348***
			(-0.498 - -0.210)	(-0.492 - -0.204)
Log GDP				0.286***
				(0.206 - 0.366)
Constant	11.93***	2.720**	2.646**	-0.150
	(10.01 - 13.86)	(0.966 - 4.475)	(0.897 - 4.396)	(-2.065 - 1.765)
**Random effects**				
Within individual				
Change rate (Age)	0.004***	0.003***	0.003***	0.003***
Intercept	2.823***	2.667***	2.593***	2.590***
Between individual				
Residuals	3.074***	3.081***	3.083***	3.084***
Observations	39,032	39,032	39,032	39,032
Number of IDs	16,027	16,027	16,027	16,027

Note: Cognitive function includes three components: immediate recall, delayed recall, and serial 7s, 0-25 points (see details in "Methods").
*** p<0.001, ** p<0.01, * p<0.05, # p<0.1

Table S5. Associations between cumulative $PM_{2.5}$ (intensity-duration) and cognitive function in multiple imputed data.

	Model 1: Basic	Model 2: Model 1 + Education	Model 3: Model 2 + SES + Marital	Model 4: Model 3 + GDP
Cumulative $PM_{2.5}$ (ref:1-1)				
1-2	-0.571**	-0.751***	-0.666***	-0.654***
	(-0.976 - -0.165)	(-1.117 - -0.385)	(-1.025 - -0.306)	(-1.012 - -0.295)
2-1	0.235	-0.0320	-0.0700	-0.0653
	(-0.0249 - 0.494)	(-0.266 - 0.202)	(-0.300 - 0.160)	(-0.295 - 0.164)
2-2	0.294**	-0.306***	-0.420***	-0.383***
	(0.0997 - 0.489)	(-0.480 - -0.132)	(-0.591 - -0.249)	(-0.554 - -0.212)
2-3	0.524***	-0.108	-0.216*	-0.224*
	(0.287 - 0.761)	(-0.327 - 0.111)	(-0.433 - -0.00015)	(-0.440 - -0.00839)
3-3	0.511***	0.0383	-0.0289	-0.0319
	(0.336 - 0.686)	(-0.117 - 0.194)	(-0.182 - 0.124)	(-0.184 - 0.121)
3-4	0.506***	-0.0706	-0.120	-0.165
	(0.315 - 0.696)	(-0.242 - 0.100)	(-0.288 - 0.0483)	(-0.334 - 0.00288)
4-4	1.011***	0.113	0.131	0.0629
	(0.780 - 1.241)	(-0.0937 - 0.319)	(-0.0711 - 0.333)	(-0.140 - 0.266)
Age	0.105***	0.237***	0.219***	0.218***
	(0.0435 - 0.167)	(0.181 - 0.292)	(0.164 - 0.274)	(0.163 - 0.273)
Age squared	-0.00222***	-0.00271***	-0.00260***	-0.00259***
	(-0.00271 - -0.0017)	(-0.0032 - -0.0023)	(-0.0030 - -0.0022)	(-0.0030 - -0.0022)
Gender (ref: men)				
Women	-1.362***	-0.115*	-0.162**	-0.185***
	(-1.476 - -1.247)	(-0.222 - -0.00776)	(-0.268 - -0.0560)	(-0.291 - -0.0791)
Education (ref: no-schooling)				
Primary		2.656***	2.432***	2.379***
		(2.524 - 2.788)	(2.301 - 2.562)	(2.248 - 2.510)
Middle		5.117***	4.362***	4.299***
		(4.970 - 5.264)	(4.206 - 4.518)	(4.143 - 4.456)
HuKou (ref: rural)				
Urban			1.227***	1.191***
			(1.091 - 1.363)	(1.054 - 1.327)
Occupation (ref: agricultural)				
Non-Agricultural			0.450***	0.421***
			(0.320 - 0.580)	(0.291 - 0.551)

Managerial			0.362***	0.350***
			(0.155 - 0.569)	(0.143 - 0.556)
Log household expenditure			0.101***	0.0984***
			(0.0787 - 0.124)	(0.0758 - 0.121)
Marital (ref: partnered)				
Single			-0.356***	-0.350***
			(-0.500 - -0.212)	(-0.494 - -0.206)
Log GDP				0.283***
				(0.203 - 0.363)
Constant	11.85***	2.728**	2.649**	-0.117
	(9.923 - 13.78)	(0.971 - 4.484)	(0.896 - 4.401)	(-2.035 - 1.800)
**Random effects**				
Within individual				
Change rate (Age)	0.004***	0.003***	0.003***	0.003***
Intercept	2.802***	2.656***	2.580***	2.577***
Between individual				
Residuals	3.074***	3.080***	3.083***	3.084***
Observations	39,032	39,032	39,032	39,032
Number of IDs	16,027	16,027	16,027	16,027

Note: Cognitive function includes three components: immediate recall, delayed recall, and serial 7s, 0-25 points (see details in "Methods"); The first number in the cumulative $PM_{2.5}$ is intensity (1: 0-35 $\mu g/m^3$; 2: 36-50 $\mu g/m^3$; 3: 51-75 $\mu g/m^3$; 4: 76+ $\mu g/m^3$), and the second represents duration (1: 0-12 months; 2: 13-60 months; 3: 61-120 months; 4: 121+ months). *** p<0.001, ** p<0.01, * p<0.05, # p<0.1

Table S6. Associations between $PM_{2.5}$ intensity (with 6 categories) and cognitive function.

	Model 1: Basic	Model 2: Model 1 + Education	Model 3: Model 2 + SES + Marital	Model 4: Model 3 + GDP
$PM_{2.5}$ intensity (ref: 0-35 $\mu g/m^3$)				
2 (36-45)	0.333**	-0.110	-0.188#	-0.148
	(0.113 - 0.552)	(-0.303 - 0.0843)	(-0.378 - 0.00244)	(-0.338 - 0.0415)
3 (46-55)	0.564***	-0.0391	-0.186*	-0.199*
	(0.353 - 0.774)	(-0.227 - 0.149)	(-0.371 - -0.00149)	(-0.383 - -0.0149)
4 (56-65)	0.421***	0.0251	-0.0141	-0.0177
	(0.220 - 0.621)	(-0.153 - 0.203)	(-0.189 - 0.160)	(-0.192 - 0.156)
5 (66-75)	0.537***	0.0173	-0.0520	-0.107
	(0.304 - 0.769)	(-0.192 - 0.227)	(-0.258 - 0.154)	(-0.313 - 0.0992)
6 (76+)	1.091***	0.198#	0.195#	0.109
	(0.839 - 1.344)	(-0.0289 - 0.424)	(-0.0278 - 0.417)	(-0.114 - 0.332)
Age	0.128***	0.249***	0.235***	0.234***
	(0.0574 - 0.199)	(0.185 - 0.313)	(0.172 - 0.299)	(0.170 - 0.297)
Age squared	-0.00232***	-0.00275***	-0.00268***	-0.00268***
	(-0.0029 - -0.0018)	(-0.0033 - -0.0023)	(-0.0032 - -0.0022)	(-0.0032 - -0.0022)
Gender (ref: men)				
Women	-1.287***	-0.0668	-0.114#	-0.141*
	(-1.416 - -1.159)	(-0.187 - 0.0538)	(-0.234 - 0.00474)	(-0.261 - -0.0224)
Education (ref: no-schooling)				
Primary		2.627***	2.418***	2.358***
		(2.477 - 2.777)	(2.269 - 2.566)	(2.209 - 2.507)
Middle		5.052***	4.323***	4.249***
		(4.886 - 5.218)	(4.147 - 4.499)	(4.073 - 4.426)
HuKou (ref: rural)				
Urban			1.169***	1.135***
			(1.014 - 1.324)	(0.980 - 1.290)
Occupation (ref: agricultural)				
Non-Agricultural			0.474***	0.437***
			(0.325 - 0.622)	(0.289 - 0.586)
Managerial			0.294*	0.273*
			(0.0627 - 0.526)	(0.0414 - 0.505)
Log household expenditure			0.0909***	0.0871***
			(0.0665 - 0.115)	(0.0628 - 0.112)
Marital (ref: partnered)				
Single			-0.353***	-0.344***
			(-0.519 - -0.186)	(-0.510 - -0.177)
Log GDP				0.343***
				(0.248 - 0.437)
Constant	10.93***	2.255*	2.195*	-1.152
	(8.738 - 13.13)	(0.251 - 4.258)	(0.195 - 4.195)	(-3.351 - 1.046)
**Random effects**				
Within individual				
Change rate (Age)	0.002***	0.004***	0.004***	0.005***
Intercept	2.864***	2.688***	2.627***	2.631***
Between individual				
Residuals	3.056***	3.062***	3.065***	3.066***
Observations	29,484	29,484	29,484	29,484
Number of IDs	12,481	12,481	12,481	12,481

Note: Cognitive function includes three components: immediate recall, delayed recall, and serial 7s, 0-25 points (see details in "Methods"); *** p<0.001, ** p<0.01, * p<0.05, # p<0.1

Table S7. Associations between $PM_{2.5}$ duration (at a threshold of 45 $\mu g/m^3$) and cognitive function.

	Model 1: Base	Model 2: Model 1 + Education	Model 3: Model 2 + SES + Marital	Model 4: Model 3 + GDP
$PM_{2.5}$ duration (ref: 1(0-12 months))				
2 (5 yrs: 13-60 months)	0.217#	-0.285**	-0.274**	-0.305**
	(-0.00765 - 0.442)	(-0.490 - -0.0807)	(-0.476 - -0.0726)	(-0.506 - -0.103)
3 (10 yrs: 61-120 months)	0.591***	0.00948	-0.0432	-0.0845
	(0.374 - 0.808)	(-0.187 - 0.206)	(-0.236 - 0.150)	(-0.278 - 0.108)
4 (10+ yrs: 121 months+)	0.587***	-0.130	-0.120	-0.214*
	(0.369 - 0.805)	(-0.327 - 0.0668)	(-0.314 - 0.0741)	(-0.409 - -0.0192)
Age	0.132***	0.259***	0.243***	0.243***
	(0.0606 - 0.203)	(0.195 - 0.323)	(0.179 - 0.306)	(0.180 - 0.306)
Age squared	-0.00236***	-0.00282***	-0.00273***	-0.00274***
	(-0.0029 - -0.0018)	(-0.0033 - -0.0023)	(-0.0032 - -0.0022)	(-0.0033 - -0.0022)
Gender (ref: men)				
Women	-1.282***	-0.0601	-0.110#	-0.138*
	(-1.411 - -1.154)	(-0.181 - 0.0604)	(-0.229 - 0.00960)	(-0.257 - -0.0190)
Education (ref: no-schooling)				
Primary		2.634***	2.422***	2.359***
		(2.484 - 2.784)	(2.274 - 2.571)	(2.211 - 2.508)
Middle		5.078***	4.354***	4.277***
		(4.913 - 5.244)	(4.178 - 4.530)	(4.100 - 4.453)
HuKou (ref: rural)				
Urban			1.139***	1.102***
			(0.984 - 1.293)	(0.947 - 1.257)
Occupation (ref: agricultural)				
Non-Agricultural			0.462***	0.423***
			(0.314 - 0.610)	(0.274 - 0.571)
Managerial			0.294*	0.271*
			(0.0618 - 0.525)	(0.0389 - 0.502)
Log household expenditure			0.0909***	0.0864***
			(0.0666 - 0.115)	(0.0620 - 0.111)
Marital (ref: partnered)				
Single			-0.357***	-0.349***
			(-0.523 - -0.190)	(-0.515 - -0.183)
Log GDP				0.370***
				(0.276 - 0.464)
Constant	10.84***	2.018*	1.983#	-1.653
	(8.636 - 13.05)	(0.00778 - 4.028)	(-0.0244 - 3.990)	(-3.860 - 0.553)
**Random effects**				
Within individual				
Change rate (Age)	0.002***	0.004***	0.004***	0.004***
Intercept	2.883***	2.690***	2.628***	2.628***
Between individual				
Residuals	3.053***	3.060***	3.063***	3.064***
Observations	29,484	29,484	29,484	29,484
Number of IDs	12,481	12,481	12,481	12,481

Note: Cognitive function includes three components: immediate recall, delayed recall, and serial 7s, 0-25 points (see details in "Methods");

*** p<0.001, ** p<0.01, * p<0.05, # p<0.1

Table S8. Associations between cumulative $PM_{2.5}$ (intensity-duration) and cognitive function (with 24 categories).

	Model 1: Basic	Model 2: Model 1 + Education	Model 3: Model 2 + SES + Marital	Model 4: Model 3 + GDP
Cumulative $PM_{2.5}$ (ref:1-1)				
1-2	0.0686	-0.260*	-0.163	-0.250#
	(-0.212 - 0.349)	(-0.519 - -0.00169)	(-0.419 - 0.0927)	(-0.507 - 0.00595)
2-2	0.240#	-0.312**	-0.318**	-0.305**
	(-0.0146 - 0.494)	(-0.539 - -0.0841)	(-0.542 - -0.0945)	(-0.528 - -0.0818)
2-3	0.866***	0.172	0.00635	-0.0203
	(0.516 - 1.216)	(-0.147 - 0.491)	(-0.309 - 0.321)	(-0.335 - 0.294)
3-3	0.595***	-0.137	-0.250*	-0.300**
	(0.354 - 0.835)	(-0.354 - 0.0794)	(-0.463 - -0.0364)	(-0.513 - -0.0868)
3-4	0.555**	-0.260	-0.299#	-0.347*
	(0.205 - 0.905)	(-0.590 - 0.0705)	(-0.627 - 0.0284)	(-0.675 - -0.0197)
4-1	0.511***	0.0746	0.0538	0.0393
	(0.267 - 0.755)	(-0.147 - 0.296)	(-0.164 - 0.272)	(-0.179 - 0.257)
4-4	0.397**	-0.210#	-0.192#	-0.252*
	(0.157 - 0.637)	(-0.428 - 0.00767)	(-0.406 - 0.0227)	(-0.467 - -0.0373)
5-4	0.564***	-0.0921	-0.121	-0.215#
	(0.305 - 0.823)	(-0.327 - 0.142)	(-0.352 - 0.110)	(-0.447 - 0.0166)
6-4	1.119***	0.0892	0.126	-0.000846
	(0.842 - 1.396)	(-0.161 - 0.339)	(-0.120 - 0.371)	(-0.248 - 0.246)
Age	0.128***	0.257***	0.242***	0.242***
	(0.0576 - 0.199)	(0.193 - 0.322)	(0.178 - 0.305)	(0.178 - 0.305)
Age squared	-0.00232***	-0.00281***	-0.00272***	-0.00273***
	(-0.00289 - -0.00176)	(-0.0033 - -0.0023)	(-0.0032 - -0.0022)	(-0.0032 - -0.0022)
Gender (ref: men)				
Women	-1.287***	-0.0627	-0.110#	-0.137*
	(-1.416 - -1.159)	(-0.183 - 0.0579)	(-0.229 - 0.00937)	(-0.257 - -0.0183)
Education (ref: no-schooling)				
Primary		2.637***	2.427***	2.366***
		(2.487 - 2.788)	(2.279 - 2.576)	(2.217 - 2.514)
Middle		5.069***	4.344***	4.271***
		(4.902 - 5.235)	(4.167 - 4.520)	(4.094 - 4.448)
HuKou (ref: rural)				
Urban			1.155***	1.118***
			(1.000 - 1.310)	(0.963 - 1.274)
Occupation (ref: agricultural)				
Non-Agricultural			0.469***	0.430***
			(0.320 - 0.617)	(0.282 - 0.579)
Managerial			0.297*	0.274*
			(0.0649 - 0.528)	(0.0421 - 0.505)
Log household expenditure			0.0896***	0.0853***
			(0.0652 - 0.114)	(0.0609 - 0.110)
Marital (ref: partnered)				
Single			-0.357***	-0.348***
			(-0.523 - -0.190)	(-0.514 - -0.182)
Log GDP				0.362***
				(0.267 - 0.457)
Constant	10.88***	2.053*	2.019*	-1.544
	(8.677 - 13.09)	(0.0407 - 4.065)	(0.0105 - 4.028)	(-3.757 - 0.668)
**Random effects**				
Within individual				
Change rate (Age)	0.002***	0.004***	0.004***	0.004***
Intercept	2.869***	2.690***	2.625***	2.628***
Between individual				
Residuals	3.054***	3.059***	3.062***	3.064***
Observations	29,484	29,484	29,484	29,484

Number of IDs	12,481	12,481	12,481	12,481

Note: Cognitive function includes three components: immediate recall, delayed recall, and serial 7s, 0-25 points (see details in "Methods"); The first number in the cumulative $PM_{2.5}$ is intensity (1: 0-35 $\mu g/m^3$; 2: 36-50 $\mu g/m^3$; 3: 51-75 $\mu g/m^3$; 4: 76+ $\mu g/m^3$), and the second represents duration (1: 0-12 months; 2: 13-60 months; 3: 61-120 months; 4: 121+ months). *** p<0.001, ** p<0.01, * p<0.05, # p<0.1

Table S9. Associations between $PM_{2.5}$ intensity, $PM_{2.5}$ intensity squared and cognitive function.

	Model 1: Basic	Model 2: Model 1 + Education	Model 3: Model 2 + SES + Marital	Model 4: Model 3 + GDP
$PM_{2.5}$ intensity				
$PM_{2.5}$	0.127	-0.0594	-0.136	-0.125
	(-0.0815 - 0.336)	(-0.244 - 0.125)	(-0.318 - 0.0455)	(-0.306 - 0.0559)
$PM_{2.5}$ squared	0.00389	0.00973	0.0169*	0.0141#
	(-0.0150 - 0.0228)	(-0.00698 - 0.026)	(0.00046 - 0.0333)	(-0.0023 - 0.0305)
Age	0.125***	0.247***	0.233***	0.232***
	(0.0546 - 0.196)	(0.183 - 0.311)	(0.170 - 0.297)	(0.169 - 0.296)
Age squared	-0.00230***	-0.00274***	-0.00267***	-0.00267***
	(-0.00287 - -0.0017)	(-0.0033 - -0.0022)	(-0.0032 - -0.0022)	(-0.0032 - -0.0022)
Gender (ref: men)				
Women	-1.285***	-0.0705	-0.120*	-0.147*
	(-1.414 - -1.157)	(-0.191 - 0.0500)	(-0.240 - -0.00134)	(-0.266 - -0.0276)
Education (ref: no-schooling)				
Primary		2.622***	2.410***	2.351***
		(2.472 - 2.772)	(2.262 - 2.558)	(2.203 - 2.500)
Middle		5.041***	4.311***	4.240***
		(4.875 - 5.206)	(4.135 - 4.487)	(4.064 - 4.417)
HuKou (ref: rural)				
Urban			1.154***	1.120***
			(0.999 - 1.308)	(0.965 - 1.275)
Occupation (ref: agricultural)				
Non-Agricultural			0.469***	0.433***
			(0.321 - 0.617)	(0.285 - 0.582)
Managerial			0.290*	0.269*
			(0.0585 - 0.522)	(0.0375 - 0.501)
Log household expenditure			0.0911***	0.0874***
			(0.0667 - 0.115)	(0.0630 - 0.112)
Marital (ref: partnered)				
Single			-0.352***	-0.343***
			(-0.518 - -0.185)	(-0.510 - -0.177)
Log GDP				0.339***
				(0.245 - 0.433)
Constant	10.69***	2.353*	2.417*	-0.904
	(8.425 - 12.95)	(0.294 - 4.413)	(0.359 - 4.476)	(-3.155 - 1.347)
**Random effects**				
Within individual				
Change rate (Age)	0.002***	0.004***	0.004***	0.005***
Intercept	2.882***	2.696***	2.632***	2.634***
Between individual				
Residuals	3.055***	3.062***	3.065***	3.066***
Observations	29,484	29,484	29,484	29,484
Number of IDs	12,481	12,481	12,481	12,481

Note: Cognitive function includes three components: immediate recall, delayed recall, and serial 7s, 0-25 points (see details in "Methods");
*** p<0.001, ** p<0.01, * p<0.05, # p<0.1

Table S10. Associations between $PM_{2.5}$ duration, $PM_{2.5}$ duration squared and cognitive function.

	Model 1: Base	Model 2: Model 1 + Education	Model 3: Model 2 + SES + Marital	Model 4: Model 3 + GDP
$PM_{2.5}$ duration				
$PM_{2.5}$	0.0522**	0.00878	-0.00782	-0.00236
	(0.0177 - 0.0867)	(-0.0227 - 0.0402)	(-0.0389 - 0.0233)	(-0.0334 - 0.0287)
$PM_{2.5}$ squared	-0.000792	-0.000303	0.000859	9.62e-05
	(-0.00277 - 0.00118)	(-0.00214 - 0.00153)	(-0.000956 - 0.0027)	(-0.00173 - 0.00192)
Age	0.121***	0.251***	0.235***	0.236***
	(0.0495 - 0.192)	(0.187 - 0.315)	(0.171 - 0.298)	(0.173 - 0.300)
Age squared	-0.00228***	-0.00277***	-0.00268***	-0.00270***
	(-0.0028 - -0.00171)	(-0.00328 - -0.00226)	(-0.00319 - -0.0022)	(-0.00321 - -0.0022)
Gender (ref: men)				
Women	-1.286***	-0.0655	-0.117#	-0.143*
	(-1.415 - -1.157)	(-0.186 - 0.0551)	(-0.236 - 0.00234)	(-0.262 - -0.0241)
Education (ref: no-schooling)				
Primary		2.626***	2.413***	2.352***
		(2.476 - 2.776)	(2.264 - 2.561)	(2.203 - 2.501)
Middle		5.058***	4.325***	4.254***
		(4.892 - 5.223)	(4.149 - 4.501)	(4.077 - 4.431)
HuKou (ref: rural)				
Urban			1.153***	1.114***
			(0.998 - 1.308)	(0.960 - 1.269)
Occupation (ref: agricultural)				
Non-Agricultural			0.466***	0.427***
			(0.318 - 0.615)	(0.279 - 0.575)
Managerial			0.288*	0.264*
			(0.0560 - 0.519)	(0.0328 - 0.496)
Log household expenditure			0.0915***	0.0877***
			(0.0671 - 0.116)	(0.0633 - 0.112)
Marital (ref: partnered)				
Single			-0.352***	-0.344***
			(-0.518 - -0.185)	(-0.510 - -0.178)
Log GDP				0.356***
				(0.261 - 0.451)
Constant	11.34***	2.161*	2.157*	-1.429
	(9.133 - 13.55)	(0.148 - 4.174)	(0.147 - 4.166)	(-3.651 - 0.793)
**Random effects**				
Within individual				
Change rate (Age)	0.002***	0.004***	0.004***	0.005***
Intercept	2.888***	2.690***	2.631***	2.631***
Between individual				
Residuals	3.056***	3.061***	3.065***	3.066***
Observations	29,484	29,484	29,484	29,484
Number of IDs	12,481	12,481	12,481	12,481

Note: Cognitive function includes three components: immediate recall, delayed recall, and serial 7s, 0-25 points (see details in “Methods”);
*** p<0.001, ** p<0.01, * p<0.05, # p<0.1

Table S11. Distribution of cumulative $PM_{2.5}$ exposure (Intensity — Duration) with three waves.

		$PM_{2.5}$ Duration (months)				Total
		1 (0-12)	2 (13-60)	3 (61-120)	4 (121+)	
$PM_{2.5}$ Intensity ($\mu g/m^3$)	1 (0-35)	7,728 (14.29)	1,092 (2.02)	0	0	8,820 (16.31)
	2 (36-50)	2,924 (5.41)	9,203 (17.02)	2,944 (5.44)	0	15,071 (27.87)
	3 (51-75)	0	169 (0.31)	16,612 (30.72)	8,472 (15.66)	25,252 (46.69)
	4 (76+)	0	0	0	4,939 (9.13)	4,939 (9.13)
Total		10,652 (19.70)	10,464 (19.35)	19,556 (36.16)	13,411 (24.80)	54,083 (100.00)

Note: Cell % in brackets; Rows are $PM_{2.5}$ duration at thresholds of 50 $\mu g/m^3$.

Table S12. Distribution of $PM_{2.5}$ exposure duration among CHARLS entrants in each wave.

	$PM_{2.5}$ exposure Duration (months)				Total
	1 (0-12)	2 (13-60)	3 (61-120)	4 (121+)	
2011 entrants	2604 (61.21)	2661 (22.31)	5364 (42.98)	1852(14.84)	12,481 (61.44)
2013 entrants	1064 (25.01)	864 (19.14)	1615 (35.78)	971 (21.51)	4514 (22.22)
2015 entrants	586 (13.78)	571 (17.21)	1075 (32.40)	1086 (32.73)	3318 (16.33)
All entrants	4254 (100.0)	4096 (100.0)	8054 (100.0)	3909 (100.0)	20,313 (100.0)

Note: Cell % in brackets; Rows are $PM_{2.5}$ duration at thresholds of 50 $\mu g/m^3$.

Table S13. Associations between cumulative $PM_{2.5}$ exposure (intensity-duration) and cognitive trajectory.

	Model 1: Base	Model 2: Model 2 + SES + Partnership	Model 3: Model 3 + GDP	Model 4: Model 3 + Interaction
**Fixed-effects coefficients**				
Cumulative $PM_{2.5}$ (ref:1-1)				
1-2	-0.612*	-0.691**	-0.684**	-0.806**
	(-1.098 - -0.125)	(-1.123 - -0.260)	(-1.115 - -0.253)	(-1.359 - -0.253)
2-1	0.394**	0.102	0.0850	0.142
	(0.0969 - 0.691)	(-0.163 - 0.366)	(-0.179 - 0.349)	(-0.196 - 0.479)
2-2	0.202#	-0.523***	-0.482***	-0.441***
	(-0.0192 - 0.423)	(-0.718 - -0.329)	(-0.676 - -0.287)	(-0.685 - -0.196)
2-3	0.522***	-0.146	-0.156	-0.141
	(0.246 - 0.797)	(-0.398 - 0.105)	(-0.408 - 0.0950)	(-0.460 - 0.178)
3-3	0.419***	-0.0886	-0.0869	0.0247
	(0.222 - 0.617)	(-0.261 - 0.0836)	(-0.259 - 0.0849)	(-0.193 - 0.243)
3-4	0.486***	-0.143	-0.195*	-0.275*
	(0.269 - 0.702)	(-0.335 - 0.0487)	(-0.388 - -0.00315)	(-0.517 - -0.0332)
4-4	1.027***	0.115	0.0359	0.249#
	(0.768 - 1.285)	(-0.112 - 0.342)	(-0.192 - 0.264)	(-0.0389 - 0.538)
Age	0.124***	0.239***	0.238***	-0.0854***
	(0.0527 - 0.194)	(0.175 - 0.302)	(0.175 - 0.302)	(-0.103 - -0.0682)
Age squared	-0.00229***	-0.00270***	-0.00271***	-0.00208**
	(-0.0029 - -0.0017)	(-0.0032 - -0.0022)	(-0.0032 - -0.0022)	(-0.0034 - -0.0008)
**Growth curve rate**				
Age # Cumulative $PM_{2.5}$ (ref:1-1)				
Age # 1-2				-0.00728
				(-0.0602 - 0.0457)
Age # 2-1				0.000782
				(-0.0292 - 0.0308)
Age # 2-2				0.00770

				(-0.0147 - 0.0301)
Age # 2-3				
				(-0.0632 - -0.0022)
Age # 3-3				0.00159
				(-0.0181 - 0.0213)
Age # 3-4				-0.0127
				(-0.0356 - 0.0102)
Age # 4-4				-0.00902
				(-0.0357 - 0.0176)
Age squared # Cumulative $PM_{2.5}$ (ref:1-1)				
Age squared # 1-2				0.00128
				(-0.0026 - 0.0052)
Age squared # 2-1				-0.000641
				(-0.0030 - 0.0018)
Age squared # 2-2				-0.000579
				(-0.0023 - 0.0011)
Age squared # 2-3				8.80e-05
				(-0.0024 - 0.0025)
Age squared # 3-3				-0.0013#
				(-0.0028 - 0.00021)
Age squared # 3-4				0.0012
				(-0.0006 - 0.0029)
Age squared # 4-4				-0.00291*
				(-0.0052 - -0.0006)
Constant	11.15***	2.132*	-1.022	3.445***
	(8.949 - 13.35)	(0.129 - 4.136)	(-3.230 - 1.186)	(2.452 - 4.439)
**Random effects**				
Between individual				
Change rate (Age)	0.003***	0.004***	0.004***	0.005***
Intercept	2.930***	2.653***	2.655***	2.667***
Covariance	0.007***	-0.010	-0.010	-0.014
Within individual				
Residuals	3.052***	3.054***	3.054***	3.063***
Observations	29,484	29,484	29,484	29,484
Number of IDs	12,481	12,481	12,481	12,481

Note: Cognitive function includes three components: immediate recall, delayed recall, and serial 7s, 0-25 points (see details in "Methods"); Model 1 only includes gender, age, age squared and cumulative $PM_{2.5}$; Model 2 controls education, household expenditure, occupations and partnership based on Model 1; Model 3 controls GDP based on Model 2; Model 4 adds the interaction between age, age squared and cumulative $PM_{2.5}$ based on Model 3; The first number in the cumulative $PM_{2.5}$ is intensity (1: 0-35 $\mu g/m^3$; 2: 36-50 $\mu g/m^3$; 3: 51-75 $\mu g/m^3$; 4: 76+ $\mu g/m^3$), and the second represents duration (1: 0-12 months; 2: 13-60 months; 3: 61-120 months; 4: 121+ months). *** p<0.001, ** p<0.01, * p<0.05, # p<0.1

Table S14. Associations between $PM_{2.5}$ intensity and cognitive function (checking age and age squared).

	Model 0	Model 1: Base	Model 2: Model 1 + Education	Model 3: Model 2 + SES + Partnership	Model 4: Model 3 + GDP
$PM_{2.5}$ intensity (ref: 0-35 $\mu g/m^3$)					
2 (36-50)	0.381***	0.374***	-0.141	-0.252**	-0.233**
	(0.180 - 0.582)	(0.173 - 0.575)	(-0.320 - 0.0373)	(-0.428 - -0.0769)	(-0.408 - -0.0577)
3 (51-75)	0.511***	0.500***	0.0363	-0.0291	-0.0443
	(0.325 - 0.696)	(0.314 - 0.685)	(-0.128 - 0.200)	(-0.190 - 0.132)	(-0.205 - 0.116)
4 (76+)	1.125***	1.087***	0.199#	0.197#	0.118
	(0.873 - 1.377)	(0.835 - 1.339)	(-0.0271 - 0.425)	(-0.0248 - 0.419)	(-0.105 - 0.340)
Age	-0.163***	0.127***	0.249***	0.236***	0.234***

	(-0.169 - -0.156)	(0.0560 - 0.197)	(0.185 - 0.313)	(0.172 - 0.299)	(0.171 - 0.297)
Age squared		-0.00231***	-0.00275***	-0.00269***	-0.00269***
		(-0.0029 - -0.0018)	(-0.0033 - -0.0023)	(-0.0032 - -0.0022)	(-0.0032 - -0.0022)
Gender (ref: men)					
Women	-1.302***	-1.286***	-0.0665	-0.116#	-0.143*
	(-1.431 - -1.174)	(-1.415 - -1.157)	(-0.187 - 0.0540)	(-0.235 - 0.00329)	(-0.262 - -0.0235)
Education (ref: no-schooling)					
Primary			2.626***	2.414***	2.356***
			(2.476 - 2.776)	(2.266 - 2.563)	(2.207 - 2.505)
Middle			5.053***	4.320***	4.247***
			(4.887 - 5.219)	(4.144 - 4.496)	(4.071 - 4.424)
HuKou (ref: rural)					
Urban				1.165***	1.130***
				(1.010 - 1.320)	(0.976 - 1.285)
Occupation (ref: agricultural)					
Non-Agricultural				0.475***	0.440***
				(0.327 - 0.623)	(0.292 - 0.588)
Managerial				0.294*	0.273*
				(0.0619 - 0.525)	(0.0410 - 0.504)
Log household expenditure				0.0912***	0.0876***
				(0.0668 - 0.116)	(0.0632 - 0.112)
Partnership (ref: partnered)					
Single				-0.349***	-0.340***
				(-0.516 - -0.183)	(-0.507 - -0.174)
Log GDP					0.332***
					(0.239 - 0.426)
Constant	19.82***	10.98***	2.265*	2.180*	-1.060
	(19.38 - 20.25)	(8.785 - 13.17)	(0.262 - 4.267)	(0.181 - 4.179)	(-3.254 - 1.135)
**Random effects**					
Within individual					
Change rate (Age)	0.003***	0.003***	0.005***	0.004***	0.004***
Intercept	2.828***	2.931***	2.797***	2.660***	2.663***
Covariance	0.008	0.014	-0.008	-0.008	-0.009
Between individual					
Residuals	3.061***	3.290***	3.051***	3.056***	3.056***
Log likelihood	-82052.296	-82019.908	-80455.387	-80218.955	-80194.873
Observations	29,484	29,484	29,484	29,484	29,484
Number of IDs	12,481	12,481	12,481	12,481	12,481

Note: Cognitive function includes three components: immediate recall, delayed recall, and serial 7s, 0-25 points (see details in "Methods");
*** p<0.001, ** p<0.01, * p<0.05, # p<0.1

73

Figures

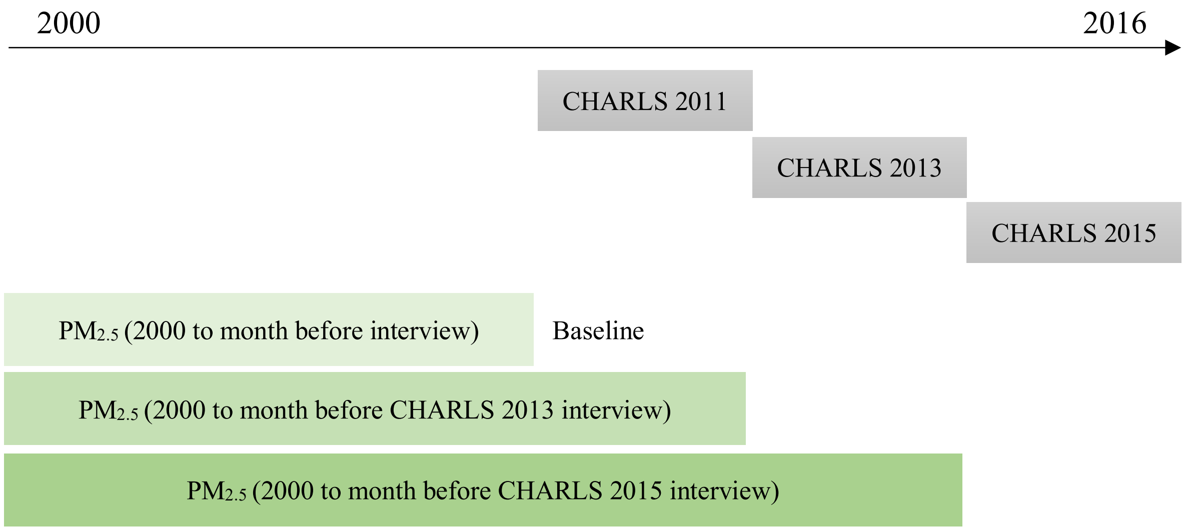

Figure S1. $PM_{2.5}$ exposure and CHARLS periods. Due to the varying interview date of each respondent, the period of exposure is measured from March of 2000 to the month preceding cognitive assessment in each wave of CHARLS.

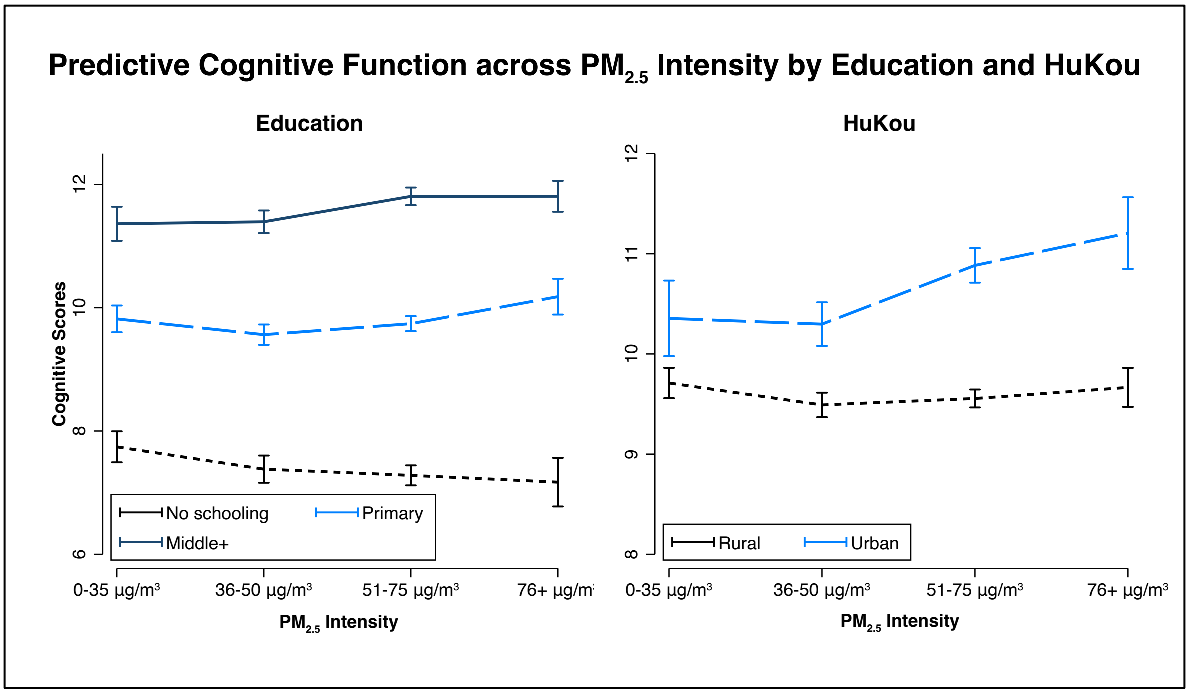

Figure S2. Associations between $PM_{2.5}$ intensity and cognitive function by SES groups (education and HuKou). Adjusted covariates includes age, age squared, gender, HuKou/education, occupation, household expenditure (logged), and annual GDP at the city level (logged).

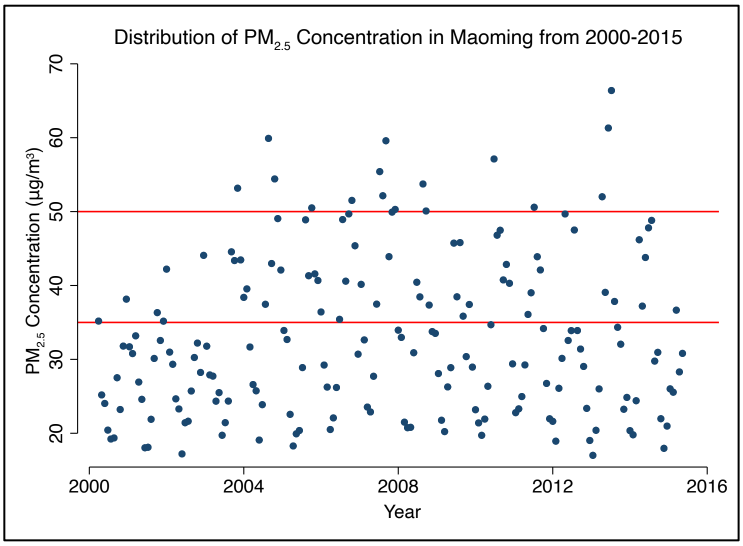

Figure S3. Distribution of monthly average of $PM_{2.5}$ concentration in Maoming city from 2000-2015.

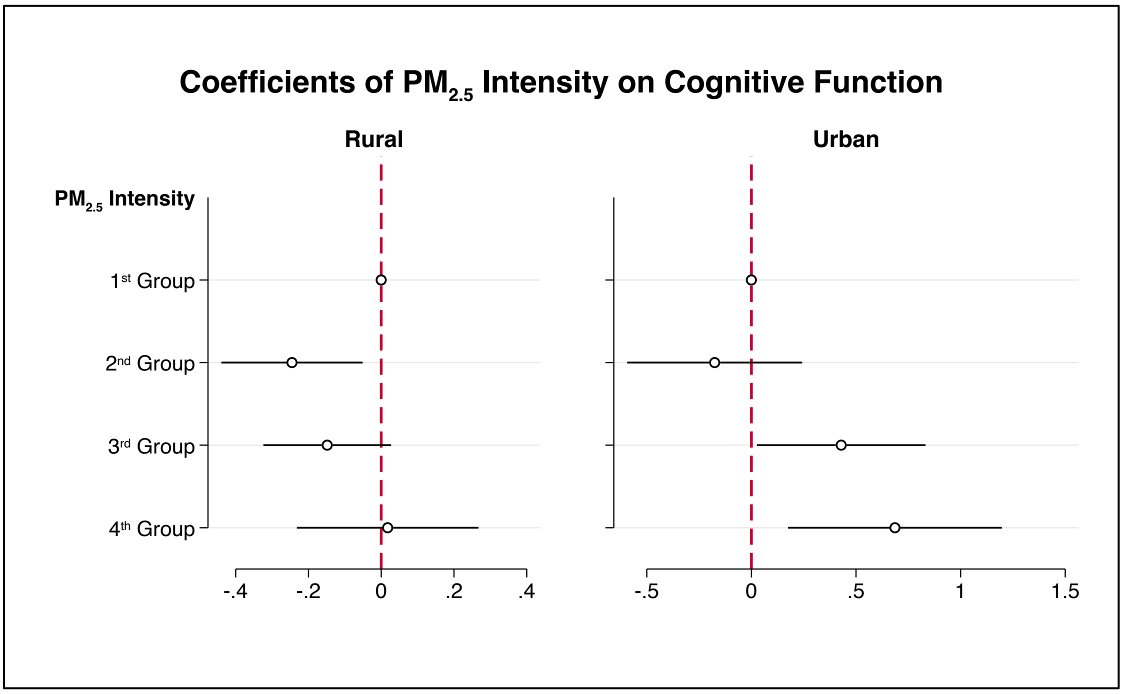

Figure S4. Associations between $PM_{2.5}$ intensity and cognitive function stratified by HuKou status. Adjusted covariates include age, age squared, gender, education, HuKou, occupation, household expenditure (logged), and annual GDP at the city level (logged). Note: 1st Group (0-35 $\mu g/m^3$); 2nd Group (36-50 $\mu g/m^3$); 3rd Group (51-75 $\mu g/m^3$); 4th Group (76+ $\mu g/m^3$).

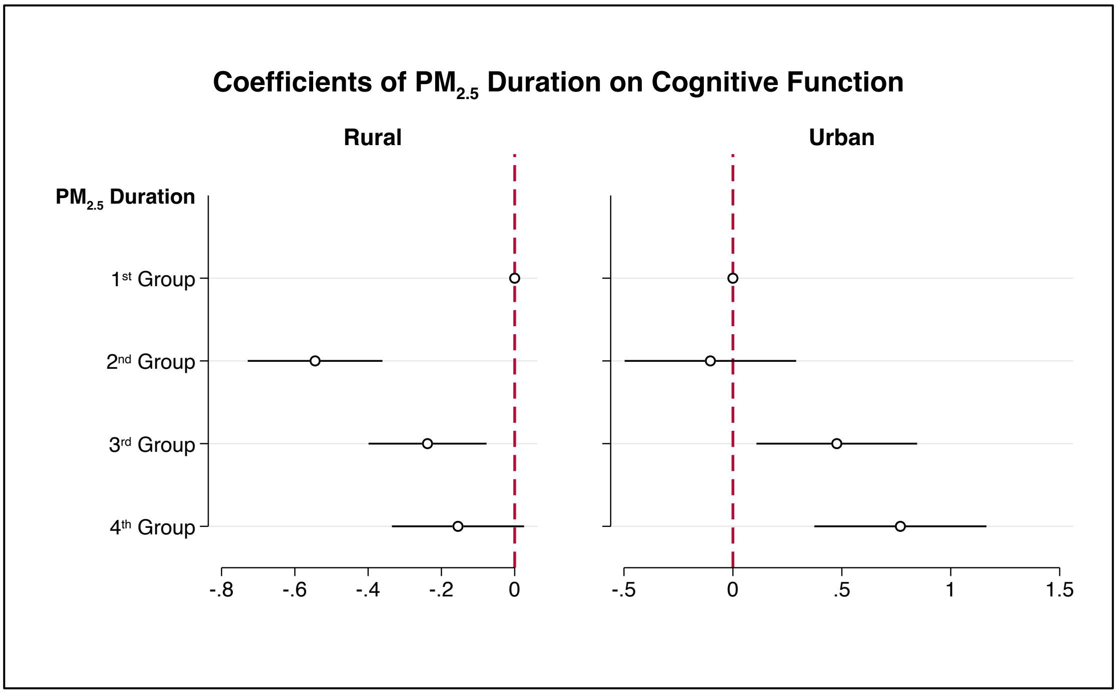

Figure S5. Associations between $PM_{2.5}$ duration and cognitive function stratified by HuKou status. Adjusted covariates include age, age squared, gender, education, HuKou, occupation, household expenditure (logged), and annual GDP at the city level (logged). Note: 1st Group (0-12 months); 2nd Group (13-60 months); 3rd Group (61-120 months); 4th Group (121+ months).

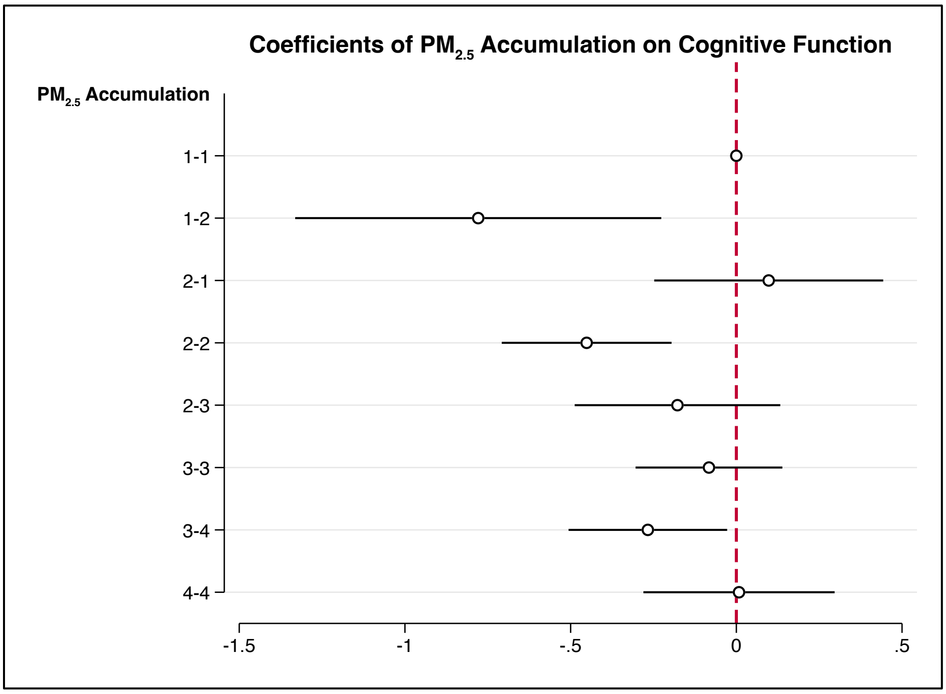

Figure S6. Associations between cumulative $PM_{2.5}$ exposure (intensity-duration) and cognitive function in the balanced data. Adjusted covariates include age, age squared, gender, education, HuKou, occupation, household expenditure (logged), and annual GDP at the city level (logged). The first number in the cumulative $PM_{2.5}$ is intensity (1: 0-35 μg/m^3; 2: 36-50 μg/m^3; 3: 51-75 μg/m^3; 4: 76+ μg/m^3), and the second represents duration (1: 0-12 months; 2: 13-60 months; 3: 61-120 months; 4: 121+ months).

References

Carpenter, J.R., Kenward, M.G., 2013. Multiple Imputation and its Application, Multiple Imputation and its Application. John Wiley & Sons, Ltd, Chichester, UK. https://doi.org/10.1002/9781119942283

Fisher, G.G., Hassan, H., Rodgers, W.L., Weir, D.R., Arbor, A., 2013. Health and Retirement Study Imputation of Cognitive Functioning Measures: (Final Release Version) Data Description.

Mccammon, R.J., Fisher, G.G., Hassan, H., Faul, J.D., Rodgers, W.L., Weir, D.R., 2019. Health and Retirement Study Imputation of Cognitive Functioning Measures: 1992-2016 (Version 1.0) Data Description.

Rubin, D.B., 1976. Inference and Missing Data. Biometrika 63, 581. https://doi.org/10.2307/2335739

White, I.R., Royston, P., Wood, A.M., 2011. Multiple imputation using chained equations: Issues and guidance for practice. Statistics in Medicine 30, 377–399. https://doi.org/10.1002/sim.4067
